# BH3-only sensors Bad, Noxa and Puma are Key Regulators of Tacaribe virus-induced Apoptosis

**DOI:** 10.1371/journal.ppat.1008948

**Published:** 2020-10-12

**Authors:** Julia Holzerland, Lucie Fénéant, Logan Banadyga, Julia E. Hölper, Michael R. Knittler, Allison Groseth

**Affiliations:** 1 Junior Research Group Arenavirus Biology, Friedrich-Loeffler-Institut, Federal Research Institute of Animal Health, Greifswald—Isle of Riems, Germany; 2 Special Pathogens Program, National Microbiology Laboratory, Public Health Agency of Canada, Winnipeg, MB, Canada; 3 Institute of Molecular Virology and Cell Biology, Friedrich-Loeffler-Institut, Federal Research Institute of Animal Health, Greifswald—Isle of Riems, Germany; 4 Institute of Immunology, Friedrich-Loeffler-Institut, Federal Research Institute of Animal Health, Greifswald—Isle of Riems, Germany; Division of Clinical Research, UNITED STATES

## Abstract

Pathogenicity often differs dramatically among even closely related arenavirus species. For instance, Junín virus (JUNV), the causative agent of Argentine hemorrhagic fever (AHF), is closely related to Tacaribe virus (TCRV), which is normally avirulent in humans. While little is known about how host cell pathways are regulated in response to arenavirus infection, or how this contributes to virulence, these two viruses have been found to differ markedly in their ability to induce apoptosis. However, details of the mechanism(s) governing the apoptotic response to arenavirus infections are unknown. Here we confirm that TCRV-induced apoptosis is mitochondria-regulated, with associated canonical hallmarks of the intrinsic apoptotic pathway, and go on to identify the pro- and anti-apoptotic Bcl-2 factors responsible for regulating this process. In particular, levels of the pro-apoptotic BH3-only proteins Noxa and Puma, as well as their canonical transcription factor p53, were strongly increased. Interestingly, TCRV infection also led to the accumulation of the inactive phosphorylated form of another pro-apoptotic BH3-only protein, Bad (i.e. as phospho-Bad). Knockout of Noxa or Puma suppressed apoptosis in response to TCRV infection, whereas silencing of Bad increased apoptosis, confirming that these factors are key regulators of apoptosis induction in response to TCRV infection. Further, we found that while the highly pathogenic JUNV does not induce caspase activation, it still activated upstream pro-apoptotic factors, consistent with current models suggesting that JUNV evades apoptosis by interfering with caspase activation through a nucleoprotein-mediated decoy function. This new mechanistic insight into the role that individual BH3-only proteins and their regulation play in controlling apoptotic fate in arenavirus-infected cells provides an important experimental framework for future studies aimed at dissecting differences in the apoptotic responses between arenaviruses, their connection to other cell signaling events and ultimately the relationship of these processes to pathogenesis.

## Author summary

Arenaviruses are important zoonotic pathogens that present a serious threat to human health. While some virus species cause severe disease, resulting in hemorrhagic fever and/or neurological symptoms, other closely related species exhibit little or no pathogenicity. The basis for these dramatically different outcomes is insufficiently understood, but investigations of host cell responses have suggested that apoptosis, i.e. non-inflammatory programmed cell death, is regulated differently between pathogenic and apathogenic arenaviruses. However, many questions remain regarding how these viruses interact with cell death pathways upon infection. Here we demonstrate that apoptosis induced by the avirulent Tacaribe virus (TCRV), proceeds via the mitochondria (i.e. the intrinsic apoptotic signaling pathway), and is regulated by a combination of factors that appear to balance activation (i.e. Noxa and Puma) and inactivation (i.e. Bad-P) of this cascade. During TCRV infection, the balance of these pro- and anti-apoptotic signals shifts the equilibrium late in the infection towards cell death. Importantly, we also found that the highly pathogenic Junín virus (JUNV), which does not trigger caspase activation or apoptotic cell death, nonetheless induces pro-apoptotic factors, thus supporting the existence of a specific mechanism by which this virus is able to evade apoptosis at late stages in this process.

## Introduction

The arenavirus family is made up mainly of rodent-borne viruses, although exclusively reptile-associated viruses have also recently been identified [1]. The classical mammalian arenaviruses are further sub-classified according to their antigenic properties, geographical distribution and phylogeny into the Old World (OW) and New World (NW) arenaviruses [2, 3]. Regardless, they all share a common structure consisting of an enveloped particle containing a bi-segmented negative strand RNA genome, with both segments encoding two open reading frames (ORFs) in an ambisense coding arrangement [4]. The small genome segment (S segment) encodes the glycoprotein (GP) and the nucleoprotein (NP), while the large genome segment (L segment) encodes the polymerase (L) and the matrix protein (Z) [5].

Although arenaviruses possess such a limited coding capacity, they include a number of notable human pathogens. For instance, several NW arenaviruses cause severe hemorrhagic fever (HF) diseases with neurological manifestations in geographically restricted regions of South America. The best studied of these viruses is Junín virus (JUNV), which is endemic to agricultural regions of central Argentina and causes Argentine Hemorrhagic Fever [6–9]. In contrast, the closely related Tacaribe virus (TCRV) is not known to cause human disease in a natural setting, although it appears to be able to infect humans, as indicated by rare symptomatic laboratory acquired infections [10]. At present, the basis for these differences in pathogenicity remains unclear, although it is likely that differences in the ability of virulent and avirulent arenaviruses to modulate host cell responses play a role. Current evidence suggests that arenaviruses regulate both type I interferon (IFN-I) production and nuclear factor-κB (NFκB) signaling via inhibitory interactions of NP and Z with host components of these signaling pathways (reviewed in [11]). There is also evidence that regulation of apoptosis by arenaviruses may play an important role in infection and be linked to pathogenesis (reviewed in [12]).

**Fig 1:**
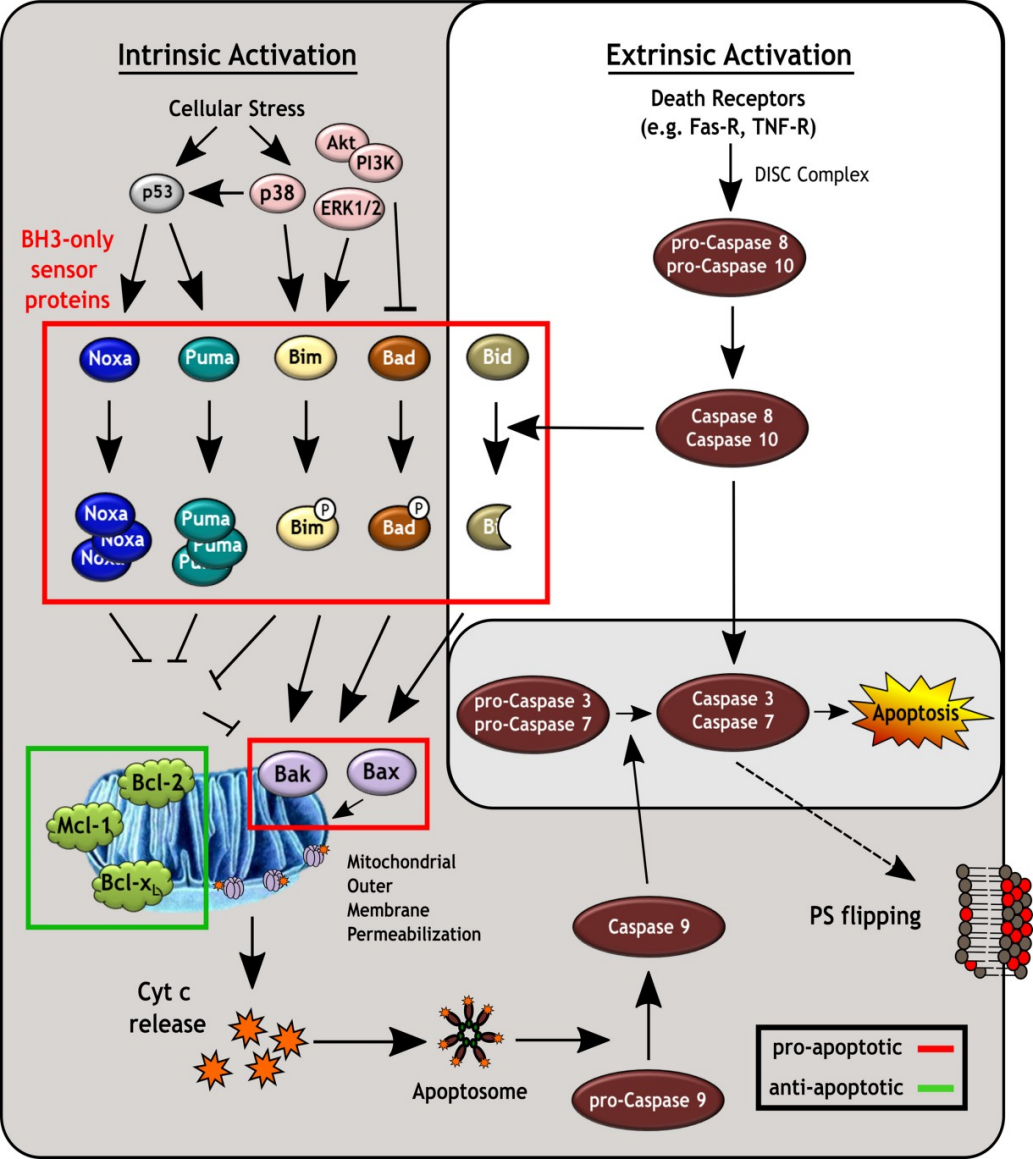
Apoptosis induction via the extrinsic and intrinsic pathways. The extrinsic activation of apoptosis proceeds via binding of pro-apoptotic ligands, such as FasL, TRAIL or TNF-α to their corresponding “death receptors” and mediates the formation of the death-inducing signaling complex (DISC) in conjunction with initiator caspases 8 and/or 10. This in turn activates these caspases, and leads to subsequent activation of effector caspases 3 and/or 7. In contrast, intrinsic activation occurs via distinct signal transduction pathways and can be triggered by a variety of cellular stressors (e.g. reactive oxygen species, ER stress, DNA damage), which are frequently recognized by cellular kinases (e.g. p38, Akt, PI3K, ERK1/2). These signaling molecules can then transmit “danger signals” either directly to pro-apoptotic BH3-only proteins, or via related regulatory factors, such as p53. Once activated, BH3-only proteins promote mitochondrial outer membrane permeabilization by activating Bak/Bax oligomerization either directly or indirectly (i.e. by antagonizing anti-apoptotic Bcl-2 proteins). The resulting oligomerization of Bak and/or Bax leads to the formation of pore structures through which Cyt c escapes to form the apoptosome (together with Apaf-1), which then activates initiator caspase 9 and subsequently the effector caspases 3 and/or 7. Caspase activity leads to the degradation of a variety of host protein components, ultimately leading to cell death as well as to the irreversible presentation of phosphatidylserine (PS) on the plasma membrane for clearance of apoptotic cells. Pro-apoptotic factors are framed by red boxes and anti-apoptotic factors are framed by a green box.

Apoptosis is a non-inflammatory programmed cell death process that leads to the fragmentation of the cell and the release of membrane enclosed cell remnants (apoptotic bodies) that are enriched for phosphatidylserine (PS), thus making them targets for clearance by phagocytic cells through PS-receptors (reviewed in [13]). A further defining hallmark of apoptosis is that it involves the orderly degradation of host cell components by cysteine-aspartic proteases (caspases; Casp), which become activated through proteolytic cleavage from an inactive proenzyme in response to cellular stress or damage. However, while many different caspases have been described, only a subset are associated with apoptosis. Among these, the initiator caspases, i.e. Casp8, 9 and 10, are mainly responsible for activation of downstream executioner caspases i.e. Casp3 and 7, which are then primarily responsible for the degradation of host cell components (reviewed in [14]; Fig 1).

While these final stages of apoptotic cell death are relatively consistent, there is a great deal of variability in how different apoptotic stimuli are sensed (reviewed in [15, 16]). The best characterized mechanisms are the extrinsic and intrinsic activation pathways (Fig 1). The extrinsic pathway activates initiator caspases (Casp8 and Casp10) in response to the activation of “death receptors” on target cells. In contrast, the intrinsic signaling pathway involves activation of initiator Casp9 in response to a variety of intracellular stresses (reviewed in [16–18]) and often involves modulation of kinase signaling pathways (reviewed in [19]). The critical checkpoint in this process is controlled by the proteins Bak and Bax, whose activation and oligomerization results in mitochondrial outer membrane permeabilization. This triggers release of Cytochrome c (Cyt c) [20–23], which is needed to form the apoptosome, and leads to initiator Casp9 activation and subsequently executioner Casp3/7 activation (reviewed in [24]). The activity of Bak/Bax is tightly regulated by a range of competing pro- and anti-apoptotic factors from within the Bcl-2 protein family. In healthy cells, the anti-apoptotic Bcl-2 proteins (e.g. Bcl-2, Bcl-x_L_, Mcl-1) inhibit activation of Bak/Bax [25–27]. The activity of these anti-apoptotic factors is, in turn, regulated by BH3-only proteins (a pro-apoptotic Bcl-2 family subset), which act as cytosolic sensors of cell stress. In response to activation they either directly activate Bak/Bax and/or antagonize the activity of the anti-apoptotic Bcl-2 proteins. Activation/inactivation of the different BH3-only proteins is complex and is controlled by a number of distinct mechanisms, including 1) regulation of protein expression levels (e.g. Puma, Noxa, Bim, Bik, Hrk), 2) sequestration on intact cytoskeletal networks (e.g. Bim, Bmf), 3) proteolytic cleavage (e.g. Bid) and 4) phosphorylation status (e.g. Bad). Ultimately it is this interplay between the pro- and anti-apoptotic Bcl-2 family members that regulates mitochondrial permeability and thus the induction of cell death through the intrinsic apoptotic pathway (reviewed in [25, 28, 29]).

To date, limited *in vitro* studies have indicated that OW arenaviruses largely avoid activating apoptosis during infection irrespective of virulence [30, 31]; although, there are some indications of apoptosis *in vivo* in neuroblasts and hepatocytes in lymphocytic choriomeningitis virus (LCMV)-infected mice [32, 33]. Although pathology data for NW arenaviruses are limited, *in vivo* evidence of apoptosis has been detected in Pichinde virus (PICV)-infected guinea pigs [34], Pirital virus-infected hamsters [35] and Machupo virus-infected macaques [36]. Further, it has recently been shown that the highly pathogenic NW arenavirus JUNV does not induce apoptosis during *in vitro* infection [37, 38], whereas the closely related but apathogenic TCRV triggers caspase-dependent apoptosis by activating Casp9 and Casp3 cleavage at late stages of infection [39]. Intriguingly, apoptosis has also recently been reported for cells infected with the attenuated vaccine strain of JUNV (Candid#1) [40–42], further highlighting the possible relevance of this process in the pathogenesis of NW arenaviruses. However, while differences in outcome at the level of cell fate have been observed in these various experimental contexts, almost nothing is known about the cellular factors that play a role in regulating this process. As a result, the possible molecular basis underlying the apparent relationship between apoptosis and pathogenesis remains enigmatic.

As a first step towards better understanding the regulation of apoptosis during arenavirus infection at the cellular level we, therefore, sought to clarify several aspects of the complex interplay between TCRV infection and the apoptotic machinery. After confirming that TCRV-induced apoptosis involves the mitochondria and proceeds via the intrinsic pathway, we further investigated the regulation of a range of pro- and anti-apoptotic Bcl-2 proteins in response to infection. In particular, we identified changes in the regulation of BH3-only proteins Bad, Noxa and Puma, as well as the transcription factor p53, both in Vero cells and in primary human monocytes, and these findings now allow us to propose a model for regulation of the pro- and anti-apoptotic balance during TCRV infection. Interestingly, infection with JUNV also revealed upregulation of p53 and Puma, thus supporting a model for JUNV infection in which pro-apoptotic signaling occurs but detectable caspase cleavage (and resulting cell death) is actively inhibited at a late stage in the apoptotic cascade.

## Results and discussion

### TCRV-induced apoptosis exhibits canonical features of the intrinsic apoptotic pathway

**Fig 2:**
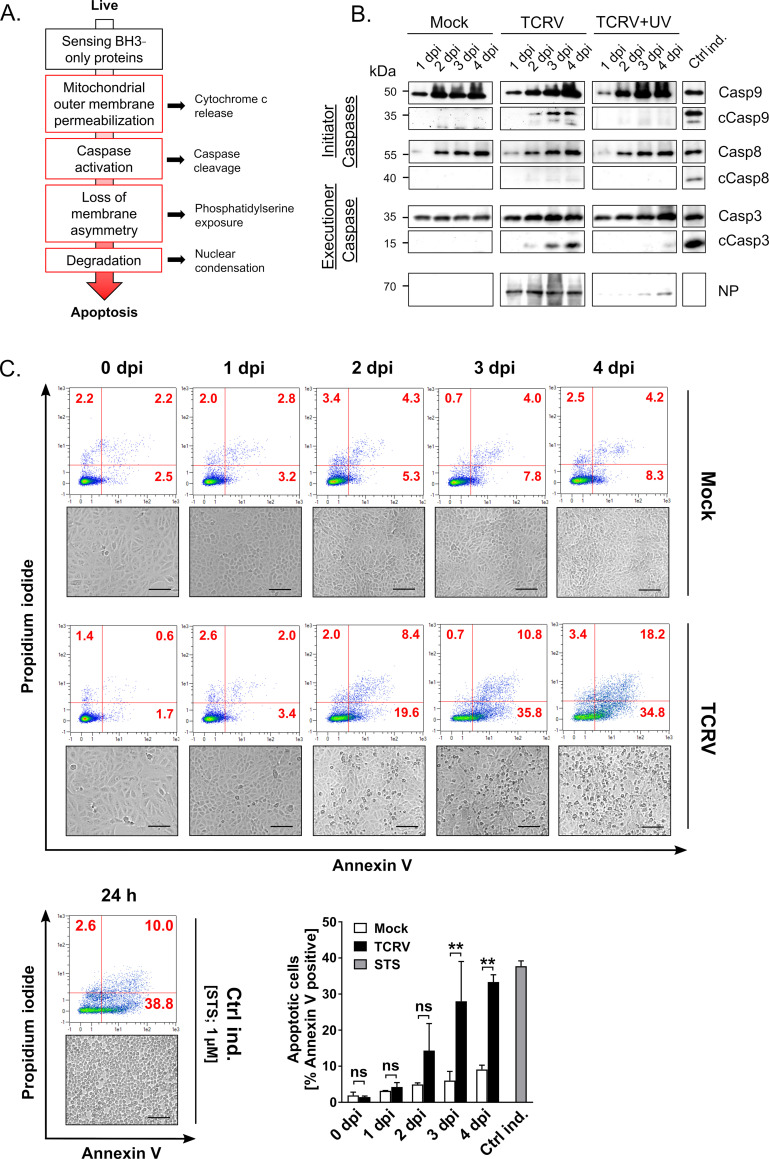
TCRV infection induces classical hallmarks of the intrinsic apoptotic pathway. (A) Schematic model of key processes in intrinsic apoptosis. Canonical events associated with intrinsic apoptosis include mitochondrial disorganization and membrane disruption, leading to Cytochrome c (Cyt c) release, caspase (Casp) activation (especially Casp9 and Casp3), phosphatidylserine (PS) flipping, and finally cell death (B) Caspase activity. Vero76 cells were mock-infected or infected (MOI = 2) with TCRV/UV-inactivated virus for 1–4 days, as indicated. Treatment with CPT (10 μM) for 24 h served as a positive control (Ctrl ind.). Cell lysates were analysed by Western blot for initiator Casp8 and Casp9, as well as executioner Casp3, using antibodies detecting both the full-length form (Casp) and cleavage products (cCasp) of the respective proteins. Detection of viral NP served as a control for infection. (C) PS flipping. Cells infected as indicated above were stained with Annexin V-FITC and PI and analysed by flow cytometry. Representative dot-plots indicate the percentage of Annexin V positive (early apoptotic cells), PI positive (necrotic cells), and Annexin V/PI double-positive cells (late apoptotic and dead cells). Treatment with STS (1 μM) for 24 h served as a positive control (Ctrl ind.). Representative bright field images of cell morphology were taken each day and are shown with 100 μm scale bars. Quantifications of PS exposure are depicted as mean values and standard deviations representing data from two independent experiments. Statistical significance was determined using two-way ANOVA (**p≤0.01, ns not significant).

TCRV infection has been previously shown to activate apoptosis-associated caspases in a variety of different cell types, with the caspases involved suggesting activation via the intrinsic apoptotic pathway (i.e. Casp3 and Casp9, but not Casp8) [39]. However, evidence for many of the canonical steps associated with intrinsic apoptosis (Fig 2A), and particularly mitochondrial involvement, has been lacking. Indeed, alternative mitochondria-independent mechanisms of Casp9 activation have also been described, and in particular can be associated with ER stress [43], which has been suggested to play a role in the induction of apoptosis by the attenuated JUNV strain Candid#1 [41, 44]. Therefore, it was deemed necessary to first better define the pathway responsible for TCRV-mediated apoptotic cell death before embarking on the identification of specific factors involved in its regulation.

We could indeed confirm prominent cleavage of initiator Casp9 and executioner Casp3, but not Casp8 in response to TCRV infection. Cleavage of Casp9 and Casp3 became visible starting 2 days after infection, increased over the course of the infection (Fig 2B), and aligned well with the development of cytopathic effect (CPE) late in infection (Fig 2C). By comparison, cells treated with UV-inactivated virus did not show cleavage of these factors, confirming that induction of apoptosis in response to TCRV relies on productive virus infection. In this context, it has been shown that overexpression of the TCRV Z protein alone can induce Casp3 cleavage [39], but the exact mechanism(s) by which it does so, and whether this is reflective of the mechanism driving apoptosis induction during an infection remain to be investigated. Nonetheless, the observation that apoptotic activation appears to occur in some experiments only well after a robust infection, as indicated by NP accumulation, is established, could support a role for Z as the driving force for this process consistent with the fact that Z is an antisense transcript and thus produced only late in infection. However, care needs to be taken in drawing such temporal comparisons between different antigens based on Western blotting since the antigens may have dramatically different levels of expression, and their detection relies on the use of different antibodies with different sensitivities.

Using Annexin V staining, we could also show that PS exposure on TCRV-infected cells markedly increased over the course of infection, with around 35% of the total live cell population being Annexin V positive on days 3 and 4 post infection (Fig 2C). Also a transition from an early to late apoptotic state was observed, as seen by an increase in propidium iodide (PI)/Annexin V double-positive cells, representing another 10–20% of the total live cell population, at later time points. Also the timeframe for these events was consistent with the development of CPE.

**Fig 3:**
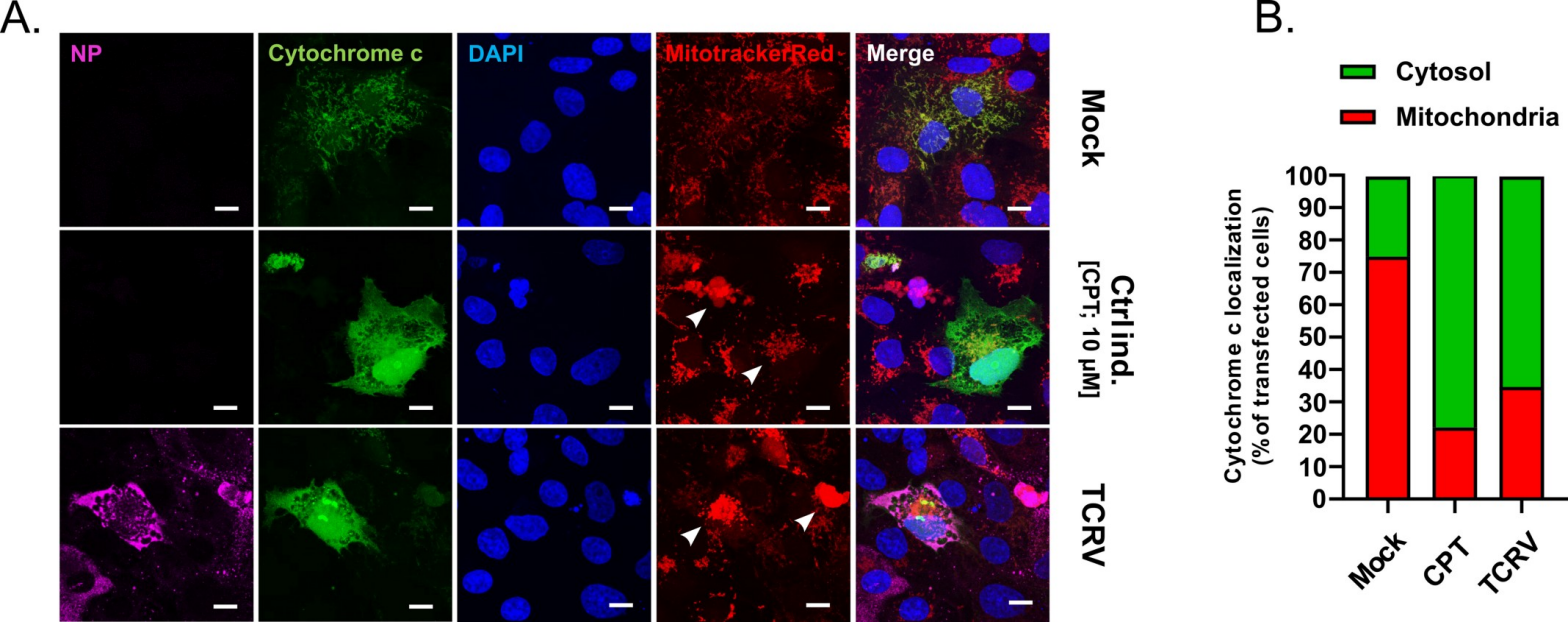
Disruption of mitochondrial networks and Cytochrome c release in TCRV-infected cells. **(A) Mitochondrial disorganization and Cytochrome c localization.** Vero76 cells transfected with a GFP fused variant of Cyt c (green), and either infected with TCRV for 3 days or treated with 10 μM CPT for 24h, before being stained with MitotrackerRed (red). Localization of NP (magenta) was detected using a guinea pig anti-TCRV NP antibody as an infection control, and nuclei were counterstained with DAPI (blue). Arrowheads indicate mitochondrial disorganization/breakdown. Bar scales show distances of 10 μm. (B) Quantification of Cyt c release. Cyt c localization was analysed for each treatment (i.e. mock, CPT-treated or TCRV-infected) based on a minimum of 200 transfected cells from randomly selected fields from two independent experiments. Localization was classified as being cytosolic (green) or mitochondrial (red) and is shown as the percentage of cells with each phenotype.

Finally, since Casp9 is classically activated in response to Cyt c release from permeabilized mitochondria, we also specifically examined infected cells for evidence of this process. While mock-treated cells showed discrete localization of Cyt c to the mitochondria, which formed well-organized mitochondrial networks, TCRV infection resulted in a subset of cells that clearly showed diffuse Cyt c staining throughout the entire cell, including in the nucleus, as well as disorganization and aggregation of the mitochondria (Fig 3A). Indeed, while some cells do appear to be infected without showing signs of Cyt c release, this is likely indicative of infection proceeding at different rates in these individual cells, and a quantification clearly shows an increase in the percentage of cells showing Cyt c release into the cytoplasm following either CPT treatment or in response to TCRV infection (Fig 3B).

Taken together, these data indicate that TCRV-induced apoptosis occurs late in the course of infection and proceeds predominantly through the intrinsic apoptotic pathway in a canonical fashion that involves dysregulation of the mitochondrial network and Cyt c release, leading to caspase 9 and 3 activation and PS exposure.

### TCRV induces upregulation of the BH3-only sensors Puma & Noxa

**Fig 4:**
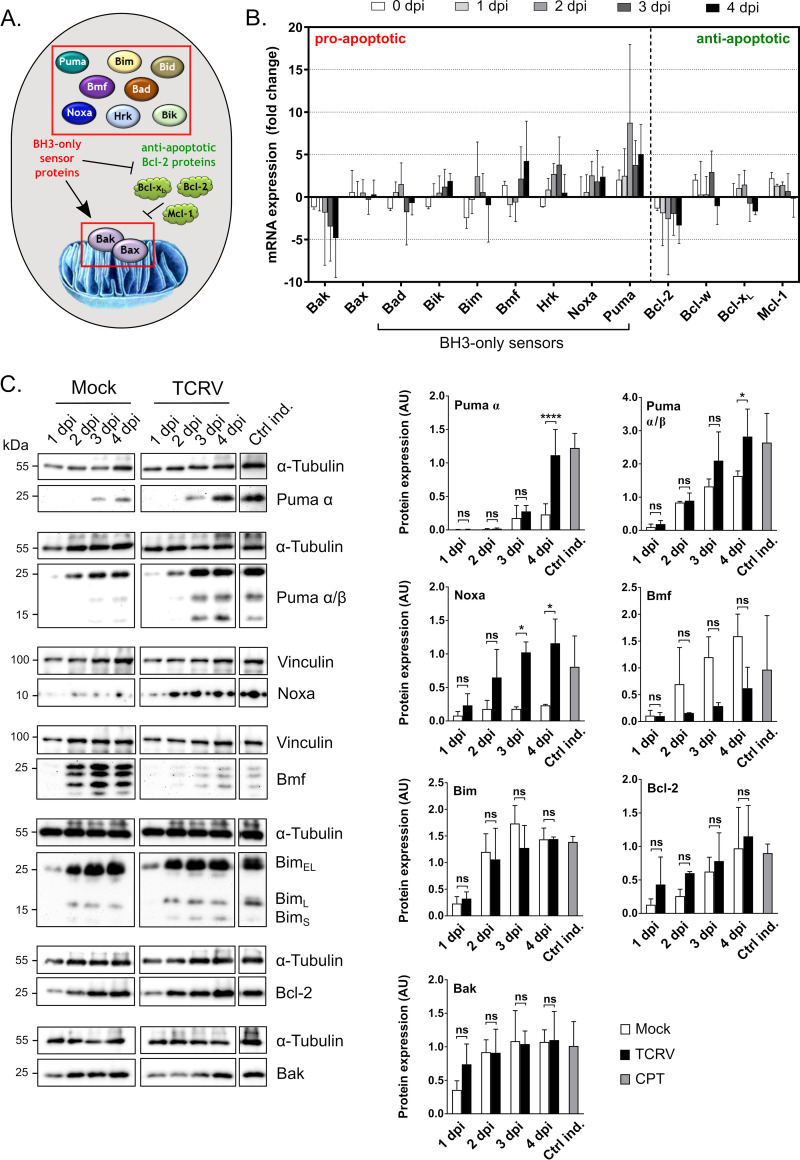
TCRV affects pro- and anti-apoptotic regulators on the mRNA transcript and protein levels. **(A) Schematic model of the host cell factors involved in regulation of mitochondrial permeability.** Pro-apoptotic BH3-only sensors can either directly activate Bak and Bax (both framed in red boxes) or antagonize their inhibitors, the anti-apoptotic Bcl-2 proteins (shown in green). **(B) Transcript levels of pro- and anti-apoptotic regulators of apoptosis.** TCRV infection was performed in Vero76 cells at an MOI of 2 and samples were harvested each day for RNA extraction. mRNA expression levels were determined using RT-qPCR with gene specific primer sets (S1 Table) from 0–4 dpi, as indicated. GAPDH levels were used for standardization and fold change in mRNA levels of TCRV-infected cells (compared to mock-infected cells) was calculated using the 2^-ΔΔCt^ method. Mean values and standard deviations of at least three independent experiments are shown. **(C) Protein levels of selected pro- and anti-apoptotic regulators of apoptosis.** Vero76 cells were infected as above and lysates were subjected to Western blotting with antibodies specific for Puma α/β, Puma α, Noxa, Bmf, Bim, Bcl-2 or Bak, as indicated. Mock cells served as a negative control, while CPT (10 μM) treated cells served as a positive control (Ctrl ind.). Staining for Vinculin or α-Tubulin, were used for loading controls. Western blots were evaluated by measuring pixel intensities for protein bands with normalization to the associated loading control. Quantifications are shown as mean values and standard deviations of at least two independent experiments. Statistical significance was determined using two-way ANOVA (*p≤0.05, ****p≤0.0001, ns not significant).

Regulation of mitochondrial permeability represents the critical checkpoint for the induction of intrinsic apoptosis. However, a mechanistic understanding of this process and the factors involved is complicated by the fact that it involves integration of input from a wide range of competing pro- and anti-apoptotic Bcl-2 family members (Fig 4A) that are themselves regulated on a variety of levels, including expression, phosphorylation state, proteolytic activation and oligomerization (reviewed in [25, 45]).

In order to gain insight into factors that might be involved in regulating TCRV-induced apoptosis, we first examined the expression levels of a variety of pro- and anti-apoptotic Bcl-2 family members using RT-qPCR (Fig 4B). Strikingly, mRNA levels for Puma (a pro-apoptotic BH3-only protein) showed markedly increased expression of almost 10-fold at 2 dpi. Other pro-apoptotic BH3-only sensor genes, such as Noxa, Bmf and Hrk, showed more modest levels of mRNA upregulation (approximately 5-fold), whereas negligible modulation of Bid, Bim, Bik and Bad mRNA levels was observed. Interestingly, mRNA levels for the pro-apoptotic factor Bak displayed a progressive reduction during infection, reaching a 5-fold decrease at 4 dpi. In contrast, expression of Bax, which plays a largely analogous pro-apoptotic role, was unaffected. Among the anti-apoptotic Bcl-2 proteins, mRNA expression for Bcl-w, Mcl-1 and Bcl-x_L_ remained unchanged, while levels of Bcl-2 were modestly downregulated.

To determine whether these changes in mRNA levels translated into meaningful changes in protein expression, we next examined protein levels for these factors using Western blot analysis. Consistent with the RT-qPCR data, the BH3-only proteins Puma (α and β isoforms) and Noxa both showed increased protein expression and accumulated during the course of the infection (Fig 4C). Upregulation of these proteins as a pro-apoptotic stimulus in the context of TCRV infection would be consistent with the current models of activation for these factors, whereby their activity is primarily governed by their expression levels, which is in turn controlled at the level of mRNA synthesis. Specifically, increased abundance of these proteins regulates the apoptotic balance by either, 1) binding anti-apoptotic Bcl-2 proteins to prevent their inhibitory association with Bak and Bax, or 2) binding of a “de-repressor” (e.g. Noxa) to the anti-apoptotic Bcl-2 proteins to prevent their inhibitory association with “direct activators” (e.g. Puma) that are then available to induce apoptosis through their association with Bak and Bax (reviewed in [25–27]). As such, the upregulation of these factors on both the mRNA and protein levels is strongly suggestive of their involvement in TCRV-mediated apoptosis.

In contrast to the mRNA expression data showing modestly increased expression at 4 dpi, Bmf, another pro-apoptotic BH3-only protein, demonstrated reduced steady-state protein levels in TCRV-infected cells (Fig 4C). Thus, it appears that other mechanisms, e.g. translational repression or increased protein turnover, may play a more significant role in regulating Bmf levels during TCRV infection. Increased mRNA expression at late time points may then suggest an auto-regulatory attempt by the cell to correct this deficiency. While initially counterintuitive to see high levels of expression of a typically pro-apoptotic BH3-only protein in healthy (mock-infected) cells, this is likely explained by the major mechanism of Bmf regulation, whereby it is normally sequestered in an inactive state by the actin cytoskeleton, but upon cytoskeleton disruption is released to promote apoptosis [46]. Indeed, similarly high basal expression has been observed in other contexts for pro-apoptotic proteins that exhibit cytoskeletal sequestration [47]. Intriguingly, this suggests that the reduced levels of Bmf observed in TCRV infected cells could impart a degree of resistance to apoptotic stimuli associated with actin cytoskeleton disruption. A similar mechanism of action is used by the BH3-only protein Bim, which is sequestered to tubulin filaments [45]; however, we saw no changes in either the mRNA or the protein expression levels of Bim, suggesting it may not play a role in apoptosis in response to TCRV-infection.

Unfortunately, for Hrk, the other BH3-only protein where mRNA expression suggested increased expression, we could not confirm protein expression since endogenous levels of this factor could not be detected under the experimental conditions using available antibodies. The same was true for Bik, although in this case mRNA expression suggested a lack of transcriptional changes.

Unlike for several of the pro-apoptotic BH3-only proteins, there appeared to be little effect of infection on levels of the anti-apoptotic Bcl-2 proteins or Bak and Bax, with most showing no change on the transcriptional level, and the modest changes suggested for Bcl-2 and Bak not corresponding to changes on the protein level (Fig 4C). Thus, it appears that the shift in the pro-apoptotic/anti-apoptotic balance during TCRV infection relies primarily on modulation of pro-apoptotic BH3-only protein expression levels, in particular Puma and Noxa, rather than changes in the abundance of anti-apoptotic Bcl-2 proteins or Bak and Bax.

### p53 is activated and undergoes nuclear translocation during TCRV infection

**Fig 5:**
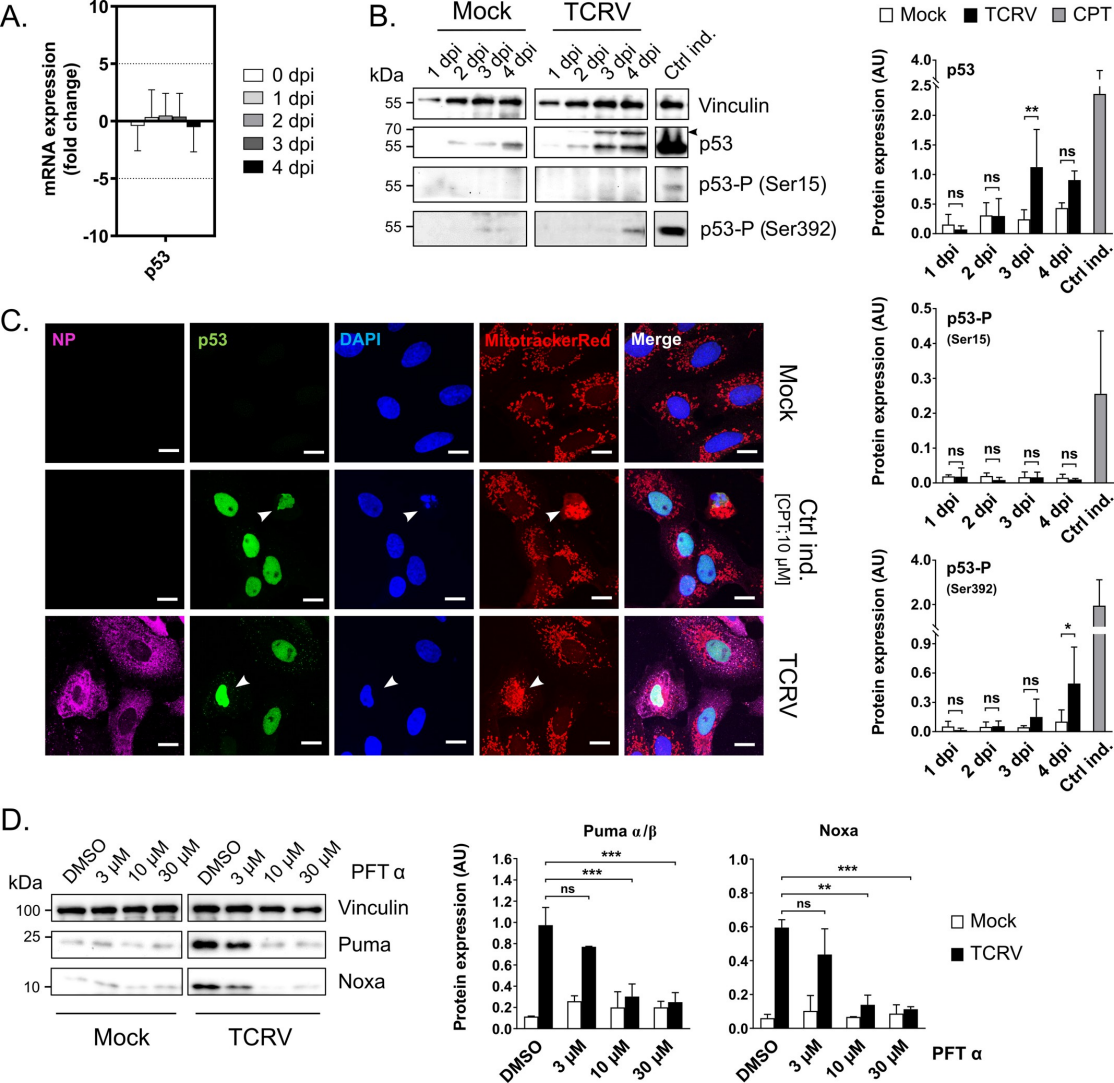
Protein expression, phosphorylation and nuclear translocation of p53 in TCRV-infected cells. **(A) Transcript levels of p53**. TCRV infection was performed in Vero76 cells at an MOI of 2 and samples were harvested each day for RNA extraction. Expression of p53 was quantified using RT-qPCR with a gene specific primer set (S1 Table) at the indicated time points between 0–4 days post infection (dpi). GAPDH levels were used for standardization and fold change in mRNA levels of TCRV-infected cells (compared to mock-infected cells) was calculated using the 2^-ΔΔCt^ method. Values represent the means and standard deviations from three independent replicates. **(B) Protein expression levels and phosphorylation of p53**. Cell lysates from TCRV-infected cells between 1 to 4 dpi were analysed by Western blot for total p53 expression, as well for phosphorylation at Ser15 or Ser392 with the respective antibodies. Vinculin served as a loading control. Mock cells served as a negative control, while CPT treatment (10 μM) was used as a positive control (Ctrl ind.). An arrowhead indicates an unidentified cross-reactive band that is also detected with the anti-p53 antibody. Western blots were evaluated by measuring pixel intensities with normalization to the associated loading control. Quantifications are based on at least two independent experiments. Statistical significance was determined using two-way ANOVA (*p≤0.05, **p≤0.01, ns not significant). (C) p53 nuclear translocation. Mitochondria were stained 3 dpi with TCRV using MitotrackerRed (red), followed by antibody staining using anti-p53 (green) and anti-TCRV NP (magenta), and labeling of nuclei with DAPI (blue). Mock cells served as a negative control, while CPT treatment (10 μM) was used as a positive control (Ctrl ind.). Arrowheads highlight morphological changes consistent with cells in the late stages of apoptosis. Scale bars show a distance of 10 μm. (D) Impact of p53 inhibition on Puma and Noxa expression. Vero76 cells were treated daily with PFT-α (3, 10 and 30 μM) or DMSO only and either Mock- or TCRV-infected (MOI = 1). Cell lysates were harvested 4 dpi and analysed for Puma and Noxa expression via Western blot, while Vinculin served as a loading control. Puma and Noxa expression were quantified based on two independent experiments and normalized to the associated loading control. Statistical significance was determined using two-way ANOVA (**p≤0.01, ***p≤0.001, ns not significant).

Given that we saw transcriptional upregulation of the BH3-only proteins Puma and Noxa, we further sought to demonstrate the activation of p53, the primary transcription factor responsible for expression of these factors. In healthy cells, the tumor suppressor protein p53 is continually degraded by the ubiquitin-proteasome pathway in a process mediated by MDM2 binding. However, stress can induce phosphorylation of p53 at multiple sites, leading to reduced MDM2-binding and stabilization of p53, which then accumulates in the nucleus where it transcriptionally upregulates a range of genes, including Puma and Noxa [48–50]. Consistent with this mechanism of activation, we saw no changes in p53 mRNA levels during the course of TCRV-infection (Fig 5A), but observed a strong increase in total p53 protein levels at 3 and 4 dpi (Fig 5B), suggesting a stabilization of the protein, rather than increased *de novo* p53 synthesis. To obtain further insight into the functional consequences of the observed increase in p53 levels, we analysed the localization of this transcription factor by immunofluorescence assay (IFA). Here we again observed enhanced expression of p53, as well as a strong accumulation of this protein within the nuclei of infected cells (Fig 5C). Some cells showing p53 translocation also showed abnormal structural morphology of the cell compartments (i.e. disorganization of mitochondria and nuclear condensation), indicating they were in the later stages of cell death. We observed similar changes when apoptosis was induced using CPT, which also results in overexpression of p53-regulated proteins, such as Puma and Noxa (c.f. control induction for these genes in Fig 4). Further, we could directly demonstrate the role of p53 in facilitating Puma and Noxa expression using a p53 inhibitor (Pifithrin-α; PFT-α), treatment with increasing concentrations of which abolished transcription of both of these factors (Fig 5D). This is then consistent with the fact that p53 is well documented to be the principle transcription factor for these genes in response to most kinds of stimuli, while Puma and Noxa are in turn responsible for the majority of the apoptotic function of p53 in many contexts (reviewed in [51]).

As noted above, the stability and activity of p53 is regulated based on a complex pattern of post-translational modifications, and in particular a number of phosphorylation sites are known to be important for its apoptotic activity (reviewed in [52, 53]). In order to gain insights into post-translational modifications associated with p53 activation in the context of TCRV infection, we used commercially available antibodies to assess its phosphorylation state. Interestingly, we did not observe phosphorylation of p53 at Ser15 in TCRV-infected cells; however, the C-terminal serine at position 392 showed clear evidence of phosphorylation at 4 dpi (Fig 5B). While phosphorylation at Ser15 has been shown to be essential for transcriptional activation by p53, this appears to require only basal levels of modification and is not necessarily inducible in response to any given apoptotic stimulus [54], as also appears to be the case for TCRV-induced p53 activation. However, modification of Ser392 alone may not be sufficient to explain the strong nuclear accumulation of p53 observed during TCRV-induced apoptosis, as phospho-Ser392 p53 has been reported to be only weakly translocated compared to other phosphorylated forms of p53 [55]. As such, this suggests that other modifications contributing to p53 stabilization and nuclear localization remain to be identified. Further, the observation that Ser392 p53 is observed only very late in comparison to the upregulation of the p53 target genes Puma and Noxa might also support a more predominant role for another modification in p53 activation, although again caution is needed in drawing temporal comparisons among different antigens. Nonetheless, taken together these results confirm that TCRV triggers modification of p53, including phosphorylation at Ser392, leading to accumulation of p53 and its recruitment into the nucleus, where it activates transcription of a gene subset that includes Puma and Noxa.

### The BH3-only sensor Bad accumulates in its inactive phosphorylated form

**Fig 6:**
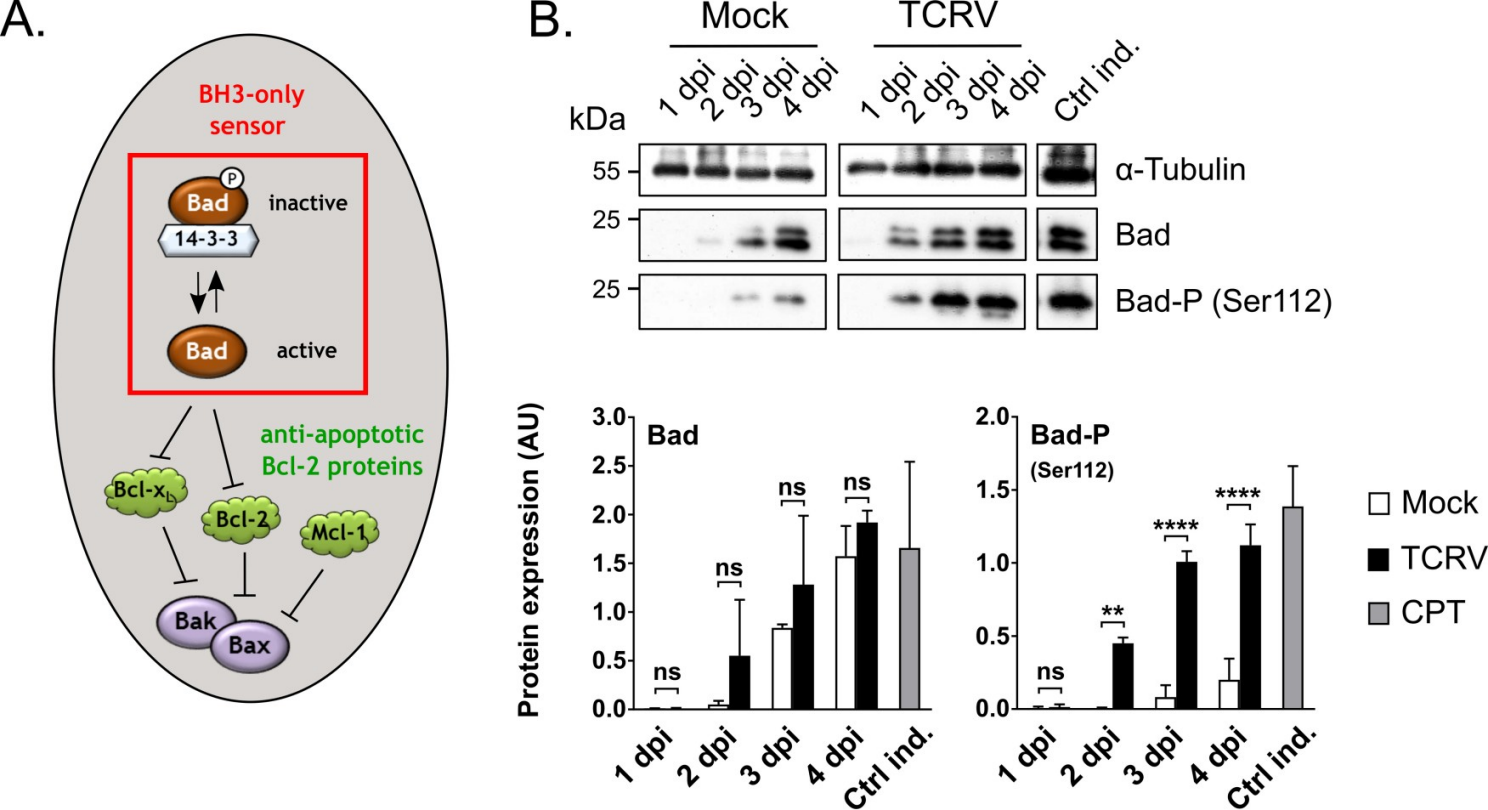
TCRV infection induces phosphorylation of the BH3-only sensor Bad. **(A) Schematic model of phosphorylation-driven regulation of Bad activity.** In its active non-phosphorylated form the pro-apoptotic BH3-only protein Bad (framed by a red box) is capable of interacting with the anti-apoptotic Bcl-2 proteins (shown in green) to lift their repression of Bak and Bax. However, in its phosphorylated state, Bad is sequestered by 14-3-3, inhibiting its pro-apoptotic activity. **(B) Protein levels and phosphorylation of Bad.** TCRV-infected and mock-infected Vero76 cell lysates were investigated at the indicated time points 1–4 dpi for total Bad, as well as phosphorylation at Ser112, with specific antibodies. Mock cells served as a negative control, while CPT treatment (10 μM) was used as a positive control (Ctrl ind.). Staining for α-Tubulin was used as a loading control. Western blots were evaluated by measuring pixel intensities and normalizing to the associated loading controls. Quantifications are shown as mean values and standard deviations of at least two independent experiments. Statistical significance was determined using two-way ANOVA (**p≤0.01, ****p≤0.0001, ns not significant).

Unlike most other BH3-only proteins associated with intrinsic apoptosis, which are primarily regulated by expression and/or sequestration, the pro-apoptotic activity of Bad is primarily regulated by phosphorylation. Phosphorylation of Bad leads to its sequestration and inactivation by 14-3-3, thereby preventing its interaction with, and inhibition of, the anti-apoptotic proteins Bcl-2 and Bcl-x_L_ [56–58] (Fig 6A). During TCRV infection, Bad showed no changes in mRNA expression level compared to mock cells (Fig 4B), and this was supported by Western blot analysis of total protein expression levels (Fig 6B). However, a strong increase was observed in the levels of Bad phosphorylated at Ser112, which is an inactive form of the protein. Accumulation of detectable levels of phospho-Bad occurred as early as 2 dpi, and increased up to 4 dpi (Fig 6B). The observation that Bad accumulates in its phosphorylated and inactivated form during TCRV infection suggests that this factor may be involved in helping to slow apoptosis induction by counteracting the other identified pro-apoptotic factors (i.e. Puma and Noxa), perhaps helping to explain why cell death during TCRV infection occurs only at comparatively late time points. Interestingly, the involvement of Bad phosphorylation in arenavirus regulation of apoptosis also suggests the association of kinase signaling pathways, several of which have been reported to be activated in response to arenavirus infection [59–68]. In particular, in some contexts Bad phosphorylation at Ser112 has been shown to rely on the ERK-dependent kinase p90-RSK [69–71], and indeed the ERK pathway is required for efficient viral replication of JUNV, TCRV and PICV [59, 60, 64, 68]. However, other kinases, such as Protein Kinase A can also mediate modification at this site [72]. As such, this topic clearly warrants further detailed investigation to establish the possible role for various kinases, and their associated signaling pathways, in the regulation of Bad (as well as p53) in response to TCRV infection.

### Bad, p53, Noxa and Puma also undergo regulation in primary human monocytes

**Fig 7:**
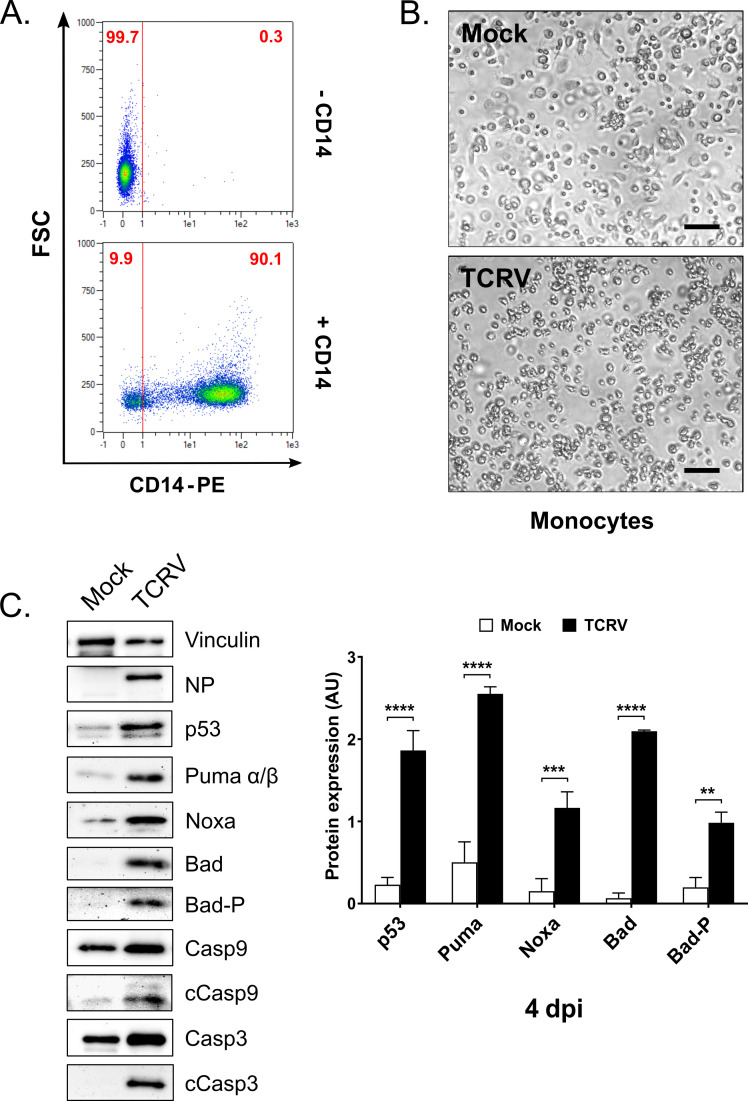
Regulation of apoptotic factors in TCRV-infected primary human monocytes. **(A) CD14 staining of isolated primary human cells.** Primary human monocyte cultures were stained with a CD14-PE antibody and analysed by flow cytometry. Representative dot-plots indicate the percentage of CD14-positive cells. **(B) Morphology of TCRV-infected monocytes.** Bright field images of mock- and TCRV-infected primary monocyte cultures were taken 4 dpi and are shown with 100 μm scale bars. **(C) Protein levels of key apoptotic factors in TCRV-infected monocytes.** Primary cells were TCRV- and mock-infected and cell lysates analysed by Western blot at 4 dpi for full-length caspase 3 and 9 (Casp3, Caps9), as well as their cleavage products (cCasp3 and cCasp9), in addition to p53, Puma, Noxa, Bad and Bad-P (Ser112). NP staining served as a positive control for infection and staining for Vinculin was used as a loading control. Quantifications show mean values and standard deviations from at least two independent experiments using different blood donors. Statistical significance was determined using two-way ANOVA (**p≤0.01, ***p≤0.001, ****p≤0.0001).

While our initial mechanistic studies of TCRV-induced apoptosis focused on Vero cells as a useful model that shows robust virus growth, and for which many assays are well established, we have previously shown that TCRV infection can activate apoptosis in a variety of different cell types [39]. However, while apoptotic pathways are highly conserved, the use of cell lines to study specific apoptotic mechanisms still presents certain risks due to the possibility of changes in expression or regulation of factors important in these pathways. In addition, due to a deletion in the relevant gene cluster, Vero cells are known to be deficient in type I interferon production [73, 74], which can also contribute to the induction of apoptosis in some contexts [75]. As such, we sought to confirm our key findings in primary human monocytes, as a biologically relevant primary cell type for arenavirus infection. For this purpose, monocytes were cultivated from peripheral blood mononuclear cells isolated from human blood and characterized by flow cytometry for CD14 expression to confirm their identity prior to use in experiments (Fig 7A). While TCRV did not produce obvious CPE during infection of monocytes (Fig 7B), the cells were productively infected and showed initiator Casp9 and executioner Casp3 cleavage (Fig 7C). Further, we detected a similar pattern of BH3-only factor activation to that identified in Vero76 cells. Specifically, we saw increased levels of p53, Puma and Noxa, as expected. Interestingly, however, expression of total Bad seemed to be more significantly increased in TCRV-infected monocytes (Fig 7C). Indeed, this provides mechanistic support for our previous observation that monocytic cells are particularly susceptible to TCRV-induced apoptosis [39], as these increased levels of Bad would be expected to provide an additional pro-apoptotic stimulus. Overall, these data suggest that the identified BH3-only factors (i.e. Puma, Noxa and Bad) are important for the regulation of TCRV-induced apoptosis in both primary and immortalized cells and that their involvement is independent of interferon production. Nonetheless, these data also indicate that there may be subtle differences between cell types in the extent to which these factors are induced and/or modified, which may then affect the susceptibility of those cell types to TCRV-induced apoptosis.

### Knockout of Noxa, Puma, and Bad modulate the induction of apoptosis during TCRV infection

**Fig 8:**
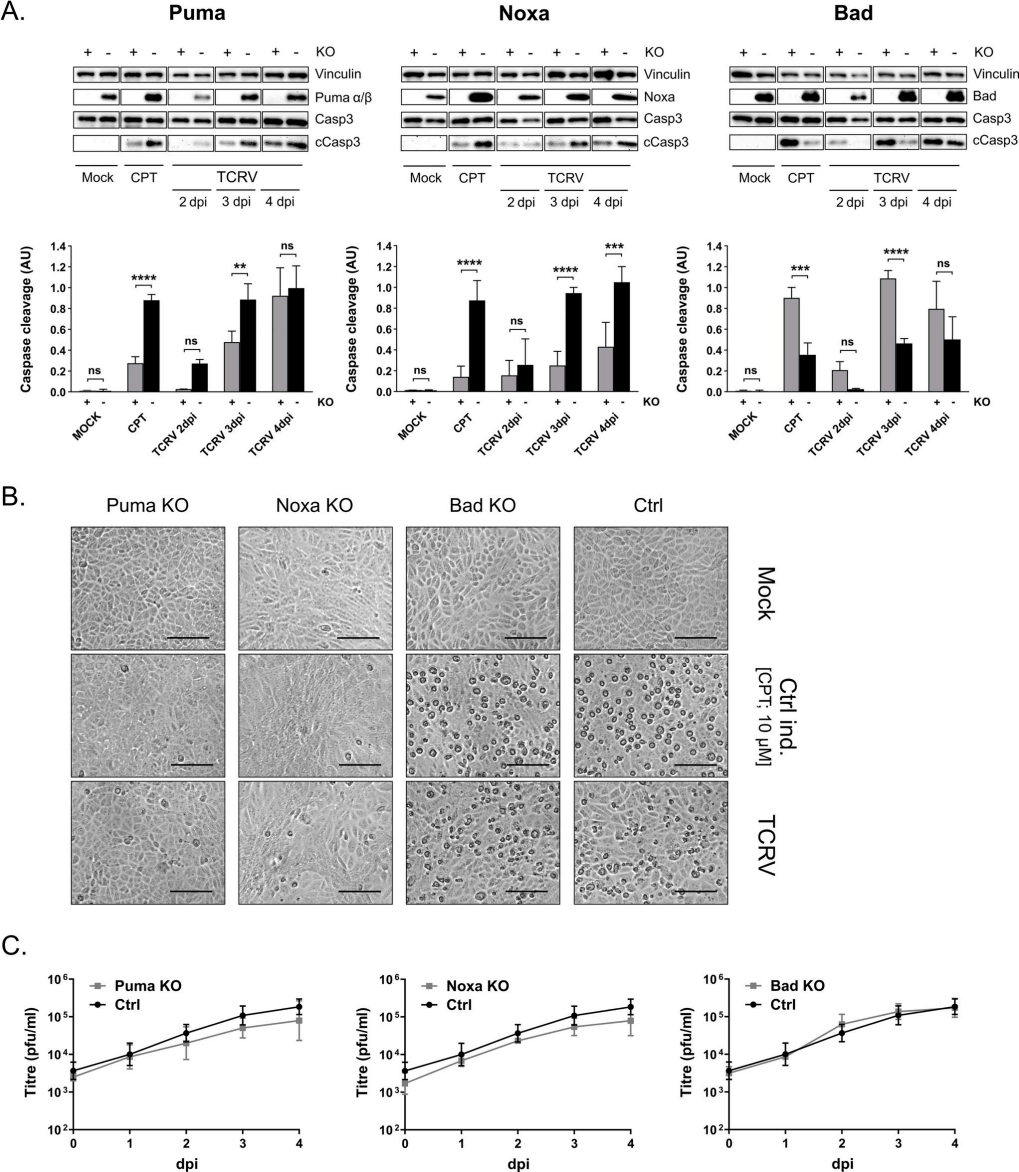
Influence of Puma, Noxa and Bad knockout (KO) on TCRV-induced apoptosis. **(A) BH3-only protein expression and caspase cleavage in KO cells infected with TCRV.** KO cells (+) and control parental cells (-) were infected with TCRV (MOI 1) or mock-infected and lysed 2 to 4 dpi before being analysed by Western blot for Bad, Noxa, Puma, full-length caspase 3 (Casp3) or its cleavage product (cCasp), as indicated, as well as the loading control Vinculin. CPT treatment (10 μM) was used as a positive control. Pixel intensities of cleaved Casp3 protein bands were measured and normalized to full-length Casp3 bands. Quantifications show mean values and standard deviations from at least two independent experiments. Statistical significance was determined using two-way ANOVA (**p≤0.01, ***p≤0.001, ****p≤0.0001, ns not significant). (B) CPE formation in KO cells during TCRV infection. Bright field images of KO and parental control cells were taken 4 dpi and are shown with 100 μm scale bars. (C) Virus growth in Bad, Noxa and Puma KO cells. Cells were infected with TCRV at an MOI of 1. Supernatants were harvested 0 to 4 dpi and virus titres were determined by plaque assay.

Given that our findings strongly suggest that TCRV-induced apoptosis is controlled to at least some extent by regulation of the pro-apoptotic BH3-only proteins Puma, Noxa and Bad, we next wanted to directly confirm their importance in this process. To facilitate these studies, we generated Puma, Noxa and Bad knockout (KO) cell lines using the CRISPR/Cas9 system. While control cells showed detectable levels of protein expression for all three factors (Bad, Puma and Noxa), analysis of the KO cells confirmed loss of the respective proteins (Fig 8A). Apoptosis induction, as demonstrated by Casp3 cleavage, was markedly reduced in Puma and Noxa KO cells during both CPT treatment and TCRV infection, although the levels of caspase cleavage still seemed to reach wild-type levels late during infection in Puma-deficient cells (i.e. 4 dpi; Fig 8A). This may be due to the effects of other BH3-only proteins, e.g. Noxa, which may compensate to some extent for the lack of Puma in these cells. The effect of these KOs is also clearly visible by observing cell morphology, where KO of either Puma or Noxa resulted in a dramatic reduction in CPE (Fig 8B). Taken together, these data support the role of Puma and Noxa as key players involved in promoting TCRV-induced apoptosis. On the other hand, KO of Bad resulted in increased apoptotic activity during TCRV infection (Fig 8A) coinciding with strong visible signs of cell death (Fig 8B). While it was surprising that loss of a pro-apoptotic factor that accumulates in its inactive form in response to infection would have a pro-apoptotic effect, this observation might be explained by resulting changes in 14-3-3 availability. As already mentioned, Bad-P is inactive due to its sequestration by 14-3-3 [56–58]; thus, the complete absence of Bad/Bad-P in our KO approach would be expected to eliminate this binding and increase the disposable amount of unbound 14-3-3 in the cell. Unbound 14-3-3 would then be available to engage in other regulatory interactions, including enhancing p53 stability/activity by binding to its negative regulator MDMX (reviewed in [76]), which would then exert a pro-apoptotic effect. Thus these findings also support a role for the accumulation of inactive phospho-Bad as an important inhibitory process that counteracts the pro-apoptotic signals generated in response to TCRV infection.

Interestingly, despite the clear connection between these individual BH3-only proteins and changes in the activation of caspase cleavage and subsequent cell death, knockout of these individual factors, whether pro- or anti-apoptotic, had little effect on virus growth *in vitro* (Fig 8C). On the one hand, this may be due to the ability of these individual knockouts to delay but not prevent caspase activation. Conversely, the lack of influence of apoptosis on virus replication may also be due to the late stage at which apoptosis occurs in response to TCRV infection, a time point by which many viral processes have already been accomplished and new virus progeny are already being efficiently produced. Thus, these data suggest that the regulation of apoptosis by TCRV may not directly support efficient virus replication and/or release at the level of individual infected cells, but rather that apoptosis induction is interconnected with the need to activate specific cell signaling pathways (such as those involving p53 and/or specific host kinases) in order to support key viral processes. Indeed, this would be consistent with a growing body of data showing that arenavirus infection can regulate and/or is dependent on a variety of kinase signaling pathways [59–68]. Alternatively, it is possible that a direct involvement of apoptosis in virus growth is only relevant in certain cell types, e.g. in macrophages where apoptotic mimicry might play an especially important role as an uptake mechanism and/or in the control of inflammatory responses. Indeed, this would potentially be supported by recent evidence that has suggested a role for PS-receptors in the entry of a number of different arenaviruses, including TCRV [77–80].

### Comparison of the activation of intrinsic apoptotic regulators during TCRV & JUNV infection

While TCRV is clearly able to induce apoptotic cell death, we have previously shown that the highly virulent JUNV evades cell death [39], presumably via the ability of JUNV NP to serve as a highly abundant decoy substrate for caspase cleavage [38]. However, given the complex network of factors that can regulate intrinsic apoptosis, we had so far been unable to experimentally validate this model. Having now established the mechanism by which TCRV triggers apoptosis, we thus also wanted to establish whether similar pro-apoptotic responses are induced in response to JUNV infection, as would be predicted by the current model.

**Fig 9:**
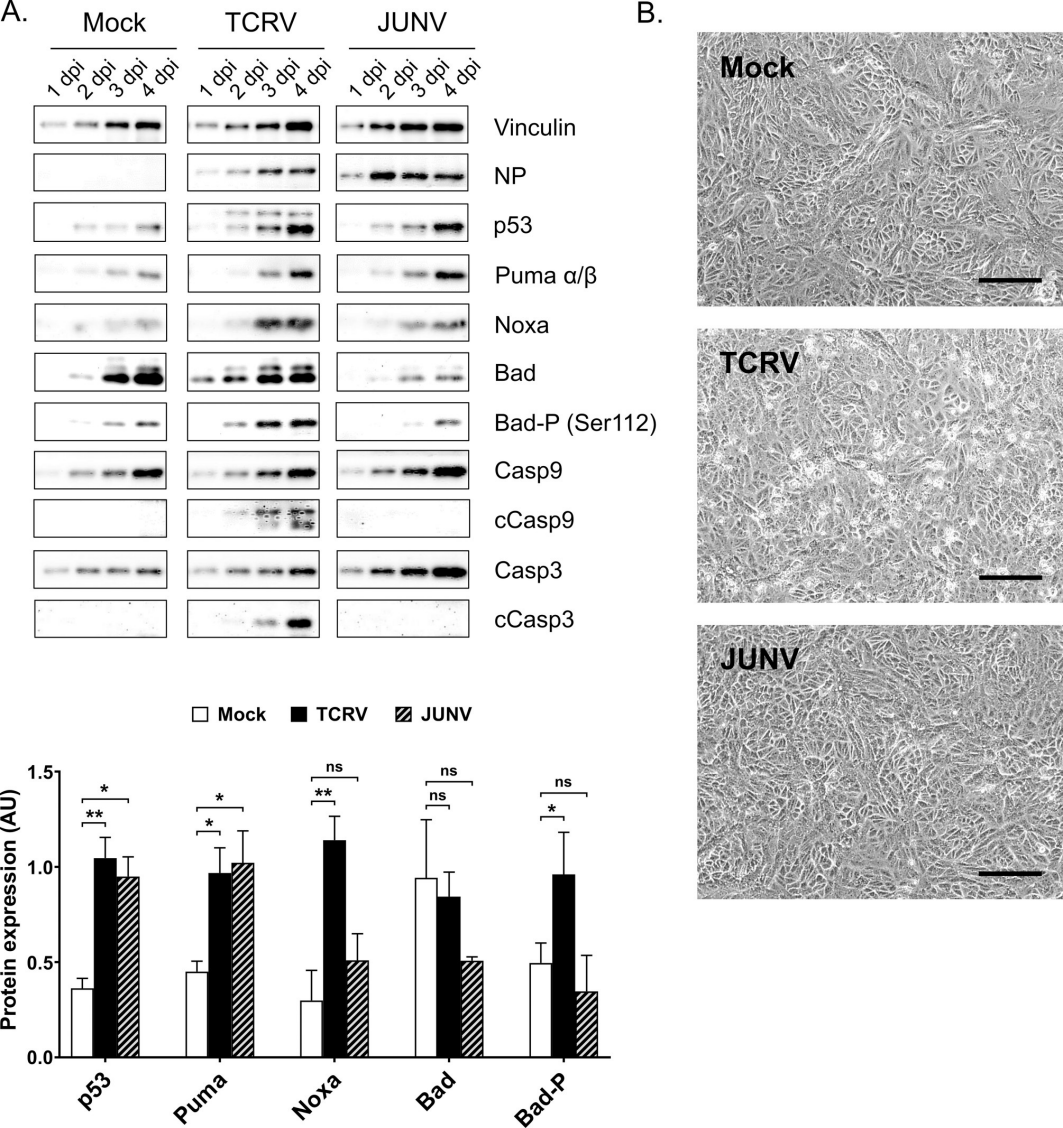
Regulation of apoptotic factors during TCRV and JUNV infection. **(A) Protein levels of selected apoptotic factors.** Infection with either TCRV or JUNV was performed at an MOI of 0.1 in Vero76 cells and cell lysates were harvested from 1–4 dpi. Samples were subjected to Western blot and stained for full-length caspase (Casp) 3 and 9, their cleavage products (cCasp), p53, Puma, Noxa, Bad and Bad-P (Ser112). NP staining served as a positive control for infection and staining for Vinculin was used as a loading control. Western blots were evaluated by measuring pixel intensities with normalization to the loading control. Quantifications of data obtained 4 dpi are shown as mean values and standard deviations of at least two independent experiments. Statistical significance was determined using two-way ANOVA (*p≤0.05, **p≤0.01, ns not significant). **(B) CPE formation in Vero76 cells during TCRV and JUNV infection.** Bright field images of mock and infected cells were taken 4 dpi and are shown with 100 μm scale bars.

Consistent with our previous findings, we confirmed that while caspase activation occurred in response to TCRV infection, there was a lack of Casp3 activation in JUNV-infected cells (Fig 9A) [38, 39]. Further, we also found that JUNV did not induce activation of the upstream initiator Casp9, indicating that the pathway is already blocked at this level. The absence of these critical late steps of apoptosis during JUNV infection is consistent with the lack of CPE seen in JUNV-infected cells (Fig 9B). Interestingly, however, analysis of the upstream BH3-only factors identified as playing a role in TCRV infection-induced apoptosis revealed that there is also marked upregulation of both p53 and Puma during JUNV infection. A slight increase in Noxa expression was also noted during JUNV infection, although this was not as robust as for TCRV (Fig 9A). These results suggest that JUNV and TCRV both induce similar pro-apoptotic signaling events at the level of BH3-only protein activation. However, unlike for TCRV, the phosphorylation of Bad appears not to be affected by JUNV infection (Fig 9A), suggesting differences in the signaling events underlying this process. Nonetheless, the observation that JUNV induces pro-apoptotic signaling yet evades caspase activation (and consequently cell death) supports the involvement of an inhibitory mechanism used by JUNV to restrict caspase activity. In particular, it would be consistent with the previously proposed model in which JUNV NP, but not TCRV NP, serves as a decoy substrate for caspases [38] to attenuate caspase cleavage and inhibit apoptosis. This then also potentially provides interesting insight into the situation with the attenuated vaccine strain of Candid#1, which unlike virulent JUNV strains induces apoptosis in response to infection [41, 42]. However, given the lack of sequence differences in NP compared to other JUNV strains [81], Candid#1 would still be expected to undergo NP cleavage by caspases. In this regard it is important to note that Candid#1 has been shown to induce apoptosis as a result of an unrelated mechanism that involves induction of the unfolded protein response due to impaired maturation of GP (as a result of a mutation affecting glycosylation) [41]. Based on our data now showing that virulent JUNV indeed induces apoptosis, before controlling it via cleavage of NP, we suggest that in the case of Candid#1, the combination of these two distinct pro-apoptotic signals, i.e. over the intrinsic pathway and the UPR pathway, may be sufficient to overwhelm the capacity of NP to compete with natural caspase substrates (including caspases themselves) in this context.

### A model for TCRV-induced apoptosis

**Fig 10:**
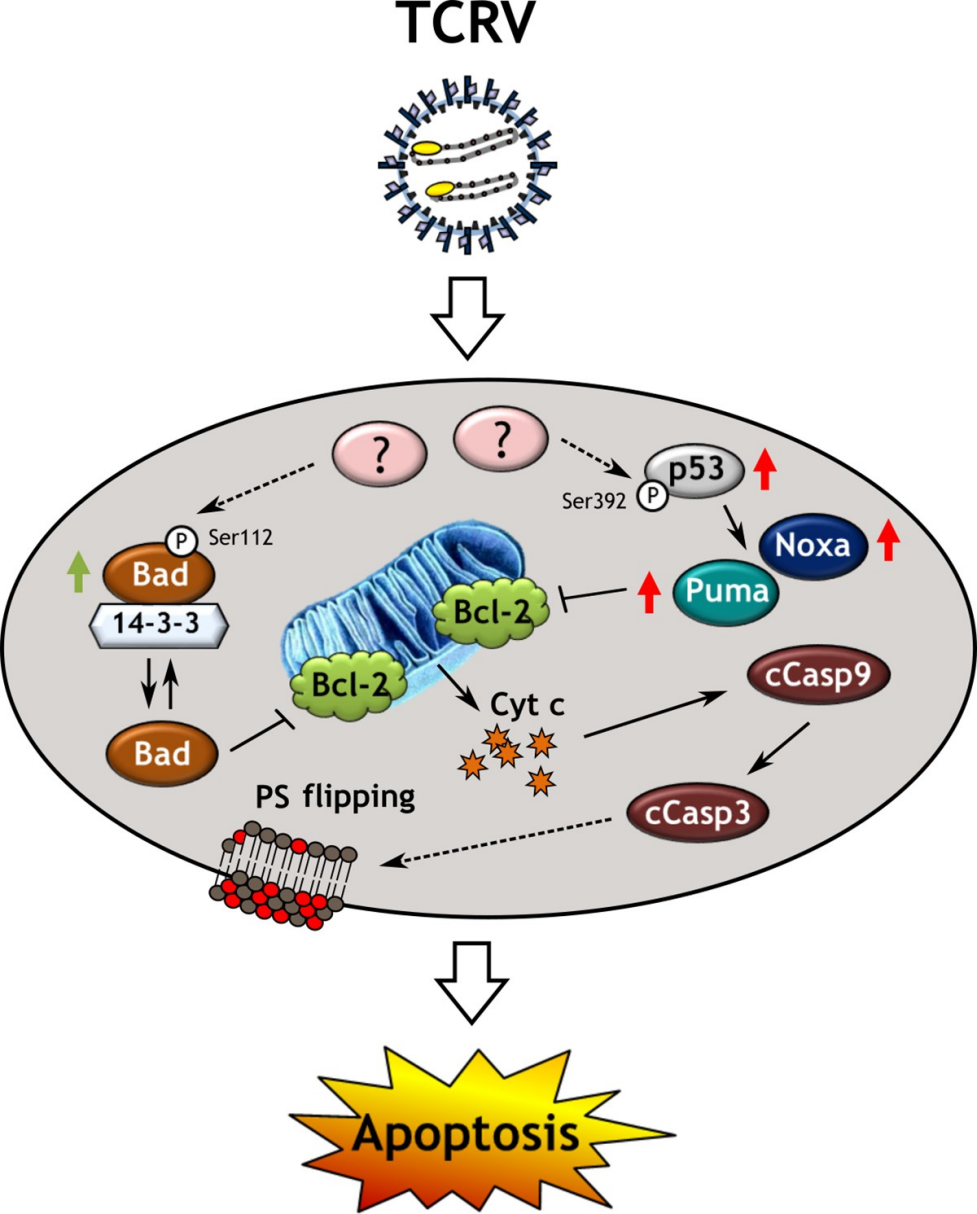
Model of intrinsic apoptotic pathway regulation by TCRV infection. The New World arenavirus Tacaribe virus (TCRV) triggers the intrinsic apoptotic pathway through regulation of the BH3-only proteins Puma, Noxa and Bad. Productive TCRV infection triggers an as yet unknown host factor/process that leads to stabilization and thus increased intracellular levels of p53, accompanied by phosphorylation at Ser392. This results in p53 nuclear translocation where it then stimulates transcriptional upregulation of the BH3-only proteins Puma and Noxa. These then bind to their antagonists, the anti-apoptotic Bcl-2 proteins, to alleviate their inhibition of Bak and Bax, and/or directly activate Bak and Bax. At the same time activation of an unidentified kinase by TCRV infection leads to phosphorylation of Bad at Ser112. This inhibits the apoptotic activity of Bad by promoting its binding to 14-3-3 to prevent its antagonistic association with anti-apoptotic Bcl-2 proteins. While the effect of Bad phosphorylation appears to counter-balance the pro-apoptotic effects of Puma and Noxa, and may delay cell death, pro-apoptotic stimuli ultimately predominate and lead to mitochondrial outer membrane permeabilization, with associated Cyt c release resulting in activating cleavage (cCasp) of initiator caspase 9 and executioner caspase 3, which then mediate apoptotic events such as PS flipping, nuclear condensation and cell death.

Overall, there is increasing appreciation that a number of viruses have developed strategies that take advantage of the apoptotic cascade in order to either evade this defensive mechanism or exploit its induction to support propagation (reviewed in [82, 83]), and our work here has led us to a model of the process by which TCRV induces apoptosis in infected cells (Fig 10). We clearly see that this virus induces apoptosis through the intrinsic apoptotic pathway and that this process is associated with classical features of mitochondrial disorganization and dysfunction, Cyt c release, initiator Casp9 and executioner Casp3 activation, PS externalization as well as nuclear condensation and eventual cell death. Further, we have shown that this process relies on upregulation of the pro-apoptotic BH3-only proteins Puma and Noxa, which are target genes of p53-mediated transcription. This enhanced transcription is facilitated by stabilization and nuclear translocation of p53, which is at least in part associated with phosphorylation of the protein at position Ser392. At the same time, TCRV also appears to induce changes that shift the pro-/anti-apoptotic balance in favor of cell survival. Notably, the normally pro-apoptotic factor Bad is converted to an inactive form by phosphorylation at position Ser112. This then results in sequestration by 14-3-3, which blocks its ability to antagonize the activity of anti-apoptotic Bcl-2 proteins [56], potentially freeing up more of these molecules to combat the activity of other pro-apoptotic BH3-only proteins induced in response to infection (i.e. Puma and Noxa). However, while these anti-apoptotic signals may allow the cell to prolong cellular survival and function, ultimately the balance of forces favors eventual apoptotic cell death at late stages of the infection.

Intriguingly, the absence of any direct effect of apoptosis on virus replication and/or release of progeny virus at the level of individual infected cells suggests a more complex basis for the association between apoptosis and pathogenesis. This now requires us to also consider processes not well represented by standard *in vitro* cell culture infection systems. In particular, a more detailed evaluation of the role of virus-containing PS-rich apoptotic bodies, as well as the effect of apoptosis-driven exposure of PS on the lipid composition of virus particles, is clearly needed. Such factors might be expected to affect recognition/uptake of these products by phagocytes and could have significant implications for the activation of antiviral immune responses in these cells, which has already been shown to differ markedly between pathogenic and apathogenic arenavirus infections [30, 84–86]. Further, it is interesting to consider how these observations may translate to regulation of cell fate in the natural host species of these viruses, which for JUNV is mice of the *Calomys spp*. (particularly *Calomys musculinus*) [6, 87] and for TCRV is currently presumed to be *Artibeus spp*. bats [88]. Indeed, based on the apparent association of apoptosis induction with pathogenesis in the human context, one might expect differences in the way a given virus is regulated in human cells and those of these host species. In this context, TCRV actually presents a particularly interesting case since, while it does not normally cause significant disease in humans, experimental infection is highly pathogenic in its presumptive host species [89]. As such, one might anticipate this being associated with an increased ability to regulate apoptotic responses in bat cells, similar to what is seen with JUNV. Unfortunately, however, the experimental resources for in depth studies in these models to determine if differential regulation of the apoptotic cascade also plays a role in the different disease outcomes between reservoir and dead-end host species are currently lacking.

Overall, this work provides us with the first detailed view of how specific pro- and anti-apoptotic factors are regulated during TCRV infection and how they contribute to virus induced apoptosis. Further, it establishes that pro-apoptotic responses are also a feature of JUNV infection, supporting a model in which caspase activation for JUNV is subsequently controlled late in the apoptotic cascade. While such studies of the role of individual BH3-only proteins in regulating apoptosis in the context of a specific virus infection remain rare, they are an important prerequisite for detailed studies aimed at unravelling the complex interplay between the various interconnected cell signaling pathways that are affected by virus infection. As such, the model generated based on this work now sets the stage for future research aimed at unraveling how closely related arenavirus species with differing pathogenic potential regulate apoptosis, as well as related pathways, and the influence this has on the outcome of infection.

## Material and methods

### Cells

Vero76 (CCLV-RIE0228) cells were grown in Dulbecco's Modified Eagle’s Medium (DMEM) supplemented with 10% fetal calf serum (FCS), 2 mM L-glutamine (Q), 100 U/mL penicillin and 100 μg/mL streptomycin (P/S; Thermo Fisher Scientific) at 37°C with 5% CO_2_.

Peripheral blood mononuclear cells (PBMCs) were isolated from whole blood of healthy anonymous donors obtained commercially from the blood bank of the Greifswald University Hospital. Samples were mixed 1:1 with PBS and layered on 15 ml of lymphocyte separation medium (1.077 g/ml Ficoll-Paque Plus, GE Healthcare). After centrifugation at 500 × g for 45 min at room temperature (RT) without brake, the PBMC layer was carefully removed and centrifuged again at 2000 × g for 20 min to isolate the cells. These were then washed twice with 15 ml PBS supplemented with 1% FCS with centrifugation at 200 × g for 10 min, before being subjected to ammonium chloride lysis (0.15 M NH_4_Cl, 10 mM KHCO_3_, 0.1 mM EDTA) for 5 min at RT to remove erythrocyte contamination. Washing with 10 ml PBS supplemented with 1% FCS was repeated until the supernatant was clear, after which the cell pellet was resuspended in RPMI-1640 with P/S and 2% human AB serum (Sigma) and seeded at a concentration of 3 x 10^6^ cells/well into Primaria 6-well plates (Corning). Cells were incubated at 37°C with 5% CO_2_ for 1 h to allow monocytes to attach and subsequently washed thoroughly three times with PBS supplemented with 1% FCS to remove lymphocytes. Afterwards, 4 ml of RPMI-1640 with P/S and 5% human AB serum was added to the cells for further incubation at 37°C with 5% CO_2_ for 1 day before use for infection and FACS analysis.

### Plasmids

Knockout of the genes for Bad, Noxa and Puma in Vero76 cells was achieved by using the CRISPR/Cas9 system. Here, a modified version of the plasmid pX330-U6-Chimeric_BB-CBh-hSpCas9 (Addgene #42230, a gift from Feng Zhang [90]) was used. This plasmid expressed the guide RNA (gRNA), trans-activating crRNA (tracrRNA) and Cas9 nuclease, as in the original construct, but with an additional neomycin resistance cassette for stable cell line selection (pX330-neoR; kindly provided by Walter Fuchs, Friedrich-Loeffler-Institut, Germany) [91]. Four different gRNAs were designed per gene, using CRISPOR [92], after which the corresponding oligonucleotides were annealed and cloned individually into pX330-neoR using BbsI. All constructs were validated by Sanger sequencing. Primers for gRNAs are listed in S1 Table and details of the cloning strategy are available upon request. A plasmid encoding an N-terminally GFP-tagged human Cytochrome c (pGFP-Cytochrome c) was a gift from Douglas Green (Addgene #41181; [93]) and was used to detect mitochondrial Cyt c release.

### Chemical induction of apoptosis and inhibition of p53

To induce apoptosis, Vero76 cells were incubated with camptothecin (CPT; 10 μM, Sigma-Aldrich), staurosporine (STS; 1 μM, Abcam) or etoposide (ET; 25 μM, Abcam) for 24 h using the indicated final concentrations in DMEM. As a control, treatment with the same amount of DMSO used to prepare the drugs was performed (S1 Fig).

To establish a direct connection between p53 activation and the upregulation of downstream targets, such as Puma and Noxa, Vero76 cells were treated with 3, 10 or 30 μM of Pifithrin-α (PFT-α), or DMSO as a control, one hour prior to TCRV infection. After infection, medium containing the same concentrations of PFT-α was applied. Treatment was refreshed daily by replacing half of the medium with fresh medium containing PFT-α and cells were harvested 4 dpi and analysed by Western Blot, as described below.

### Virus infection and plaque assay

TCRV (strain TRVL-11573) or JUNV (strain Romero) were used to infect Vero76 cells (80–90% confluence in 6- or 12-well plate formats), or primary human monocytes (prepared as described above), as indicated. Infections were carried out at a multiplicity of infection (MOI) of 0.1, 1 or 2, as indicated, and allowed to incubate for 60 min at 37°C with 5% CO_2_. Subsequently, the inoculum was removed and cells were maintained in DMEM containing 2% FCS and P/S (for Vero76 cells) or RPMI-1640 supplemented with P/S and 5% human AB serum (for human monocytes) and sampled 0 to 4 dpi, as indicated in the individual experiments. These samples were then further processed for Western blot, RT-qPCR, flow cytometry or IFA as described below.

Alternatively, in order to assess TCRV growth in knockout cell lines, the infection procedure described above was performed and supernatant samples were taken every 24 h for analysis of progeny virus release by plaque assay. Briefly, serial dilutions of virus samples were prepared in DMEM without FCS, and allowed to infect 80–90% confluent Vero76 cells in 12 well plates for 1 h at 37°C. Afterwards virus was removed and the wells overlaid with 0.7% agarose in Minimal Essential Medium (MEM) containing 2% FCS. Plates were incubated for 7 days to allow plaques to form before being stained with a crystal violet solution (10% formaldehyde; 0.1% crystal violet).

UV-inactivated TCRV was used as an additional control and was produced by inactivating the corresponding infectious TCRV stocks by irradiation at 254 nm for 2 h using a UV lamp (RU-VE CHROMA41) [94, 95], after which complete inactivation was confirmed by plaque assay, as described above.

### Antibodies

Primary antibodies obtained from Cell Signaling Technology were those against Caspase 3 (#9662; 1:1000/1:500 [full-length/cleaved]), Caspase 8 (#9746; 1:1000/1:500 [full-length/cleaved]), Caspase 9 (#9508; 1:1000/1:500 [full-length/cleaved]), α-Tubulin (#2144; 1:1000), Bim (#2933; 1:1000), Bad (#9239; 1:500), phospho-Bad (Ser112) (#5284; 1:1500), Bak (#12105; 1:1000), Noxa (#14766; 1:500) and phospho-p53 (Ser15) (#9286; 1:250), as well as the goat anti-rabbit IgG HRP-linked (#7074; 1:5000) and horse anti-mouse IgG HRP-linked (#7076; 1:5000) antibodies. The rabbit-anti-guinea pig-HRP antibody was obtained from Dianova (DAB-087875; 1:5000). Antibodies against Puma α/β (sc-374223; 1:1000), Puma α (sc-377015; 1:500), Bcl-2 (sc-7382; 1:1000), p53 (sc-47698; 1:1000; IFA—1:500), phospho-p53 (Ser392; 1:500) (sc-51690), Vinculin (sc-73614; 1:1000) and GAPDH (sc-47724; 1:2000) were obtained from Santa Cruz Biotechnology. The antibody against Bmf (HPA010120; 1:300) was obtained from Sigma-Aldrich. To confirm virus infection, polyclonal antibodies against TCRV (1:1000; IFA—1:500) and JUNV (1:1000) NP (produced in guinea pigs) were used for detection [39]. The fluorochrome conjugated goat anti-mouse IgG Alexa Fluor 488 antibody (A-11001; IFA- 1:500) was purchased from Thermo Fisher and the goat anti-guinea pig IgG Alexa Fluor 647 (ab150187; IFA- 1:500) was from Abcam. Working dilutions are indicated for Western blotting unless otherwise indicated.

### Western blot

Cells were collected in culture medium from 6- or 12-well plates by scraping, washed once with cold PBS and lysed for 40 min on ice in cell extraction buffer (CEB, Invitrogen) supplemented with 1× cOmplete Protease Inhibitor Cocktail (Sigma-Aldrich) and 1 mM Phenylmethylsulfonylfluoride (Sigma-Aldrich), followed by centrifugation at 18,000 x g for 10 minutes at 4°C. Protein lysates prepared in this fashion were then mixed with 4x SDS gel loading buffer (10% SDS (w/v), 40% glycerol (v/v), 20% β-mercaptoethanol, 0.008% Bromophenol Blue, 250 mM Tris-HCl pH 6.8) and either heated at 95°C for 5 min (for BSL-2 samples), or heated twice at 99°C for 10 min (for BSL-4 samples). Samples were separated on 12.5% polyacrylamide gels by electrophoresis and transferred to polyvinylidene difluoride (PVDF) membranes with a constant voltage of 15 V for 1 h and 15 min (Bio-Rad). Blocking was performed in 10% skim milk prepared in Tris-buffered saline containing 0.1% Tween-20 (TBS-Tween) for 60 min at RT. Primary antibodies were incubated overnight at 4°C, whereas staining with secondary antibodies was performed for 60 min at RT. In both cases antibodies were diluted in 2% skim milk in TBS-Tween as indicated. Membranes were washed thoroughly with TBS-Tween between all incubations, except for the final washing step, which was performed without Tween. ECL signals were detected using Clarity Western Substrate (Bio-Rad) using the ChemoCam Imager (Intas) and quantified using the GelAnalyzer2010 software. Data shown for each protein originate from the same gel but are shown separated by treatment condition for clarity.

### Flow cytometry

Phosphatidylserine (PS) exposure on plasma membranes was detected using the Annexin V-FITC Kit (Miltenyi Biotech). To achieve this, STS-treated, TCRV- and mock-infected Vero76 cells were harvested by trypsinization, after which fluorescein isothiocyanate (FITC) conjugated Annexin V and propidium iodide (PI) staining was performed according to the manufacturer’s protocol. Briefly, cells were washed with 1x Binding Buffer and stained with Annexin V-FITC for 15 min, followed by a further washing step and incubation with PI solution for 5 min, all performed in the dark. Without further washing, the final fixation then took place for 10 min at RT in 2% paraformaldehyde (PFA) in 1x Binding Buffer and samples were immediately measured using the MACSQuant Analyzer 10 (Miltenyi Biotech).

After attachment of human monocytes onto Primaria plates, CD14 surface expression was investigated to estimate purity of the isolated cells. Here, primary cells were detached using cold PBS supplemented with 1% FCS and centrifuged at 300 x g at 4°C for 10 min. Obtained cells were then resuspended in human FcR Blocking Reagent (Miltenyi Biotech) according to the manufacturer’s protocol and incubated for 10 min at 4°C. Subsequently, the human CD14-PE-Vio770 antibody (1:50; Miltenyi Biotech) was added and incubated in the dark on ice for a further 15 min. After another washing step, the final fixation took place for 10 min at RT in 2% PFA in PBS with FCS and samples were immediately measured using the MACSQuant Analyzer 10. Data were acquired for 20,000 cells per sample and evaluated with MACSQuantify 2.11 software (Miltenyi Biotech).

### Nucleofection

Vero76 cells were transfected by electroporation using the 4D-Nucleofector X Unit (Lonza) according to the manufacturer’s instructions. Briefly, 1×10^6^ Vero76 cells were nucleofected with a total of 5 μg of plasmid in a single Nucleovette by adding Nucleofector solution and the supplement. The recommended Program (DN-100) for Vero cells was used, and cells were subsequently seeded into 25 cm^2^ flasks (for CRISPR/Cas9 plasmid transfections) or 12 well plates containing coverslips (for pGFP-Cytochrome c transfection).

### Immunofluorescence assay

For both analysis of Cyt c localization and p53 translocation, Vero76 cells were seeded on poly-D-lysine (Sigma-Aldrich) coated microscope coverslips. In the case of Cyt c localization, cells were nucleofected with pGFP-Cytochrome c prior to seeding and infection with TCRV as described above. At 3 days post infection, cells were incubated with 200 nM MitoTrackerRed CMXRos (Invitrogen) for 40 min at 37°C and fixed with 2% PFA in PBS containing 0.5% FCS at 4°C overnight. Blocking was performed with PBS containing 10% FCS at RT for 2 h, followed by cell permeabilization in PBS containing 0.1% Triton X-100 for 10 min. Primary and secondary antibodies were also diluted in PBS containing 10% FCS and incubated for 45 min at RT before unbound antibodies were removed by washing with PBS. Finally, coverslips were mounted with ProLong Gold Antifade containing DAPI (Thermo Fisher) and cured in the dark for 24 h at RT. Microscopy was conducted with a Leica SP5 confocal laser scan microscope (63x objective) and stacks were processed with ImageJ software version 1.48 [96].

### Quantitative RT-PCR

RNA from TCRV- and mock-infected Vero76 cells was extracted with the RNeasy Mini Kit (Qiagen) and genomic DNA contamination was removed using the TURBO DNA-free Kit (Invitrogen) according to the manufacturer’s instructions. For two-step RT-qPCR, cDNA was synthesized from 400 ng total RNA with SuperScript III Reverse Transcriptase (Invitrogen), amplified by PCR using the PowerUp SYBR Green Master Mix (Thermo Fisher) and quantified with the AriaMx 96 Real-Time PCR System and AriaMx software version 1.3 (Agilent). All primers used in this study are listed in S2 Table [97–103]. Sample cycle thresholds (Ct) were normalized using GAPDH and fold change between infected and non-infected cells was calculated using the 2^-ΔΔCt^ method.

### Generation of knockout cell lines using CRISPR/Cas9

Vero76 cells were nucleofected with pX330-neoR constructs expressing gRNAs targeting Bad, Noxa or Puma as described. Cells were then allowed to recover for 3 days after which the cells were treated with DMEM containing 10% FCS, P/S and 500 μg/ml Geneticin (G418). Selection with G418 was continued for 2–3 weeks with passaging performed as needed. Each cell line was then clonally selected and expanded before being examined for their expression of Bad, Noxa and Puma by Western blot, as described above. Cell clones showing complete absence of the respective proteins were selected for use in subsequent infection experiments with TCRV. Additionally, genomic DNA from these selected clones was sequenced to confirm introduction of mutations at the expected sites.

### Statistical analysis

Quantification data represent the means and standard deviations from multiple independent experiments, as indicated for the individual assays. Statistical analysis was performed using GraphPad Prism version 8.1.0, with differences between groups analysed using two-way ANOVA with post hoc tests performed using Sidak’s test (comparison of selected pairings). Significance cut-offs were defined as * p≤0.05; ** p≤0.01; *** p≤0.001; **** p≤0.0001.

## Ethics statement

Anonymized human blood samples were obtained commercially from the blood bank of the Greifswald University Hospital with the approval of the Ethics Committee of the University of Greifswald (decision number BB 014/14).

## Supporting information

S1 Fig Testing of drugs for apoptosis control induction. In order to determine suitable doses and incubation times needed to detect caspase activation, Vero76 cells were treated with Camptothecin (CPT; 10 μM), Staurosporine (STS; 1 μM) or Etoposide (ET; 25 μM) for 24 and 48 hours. **(A) Microscopic images of induced cell death.** Morphological changes in treated cells compared to a DMSO control treatment (top picture) 48 h post treatment. Scales bars show distances of 100 μm. **(B) Detection of caspase cleavage in treated cells.** Western blot analyses were performed to investigate caspase activation using antibodies against full-length (Casp) and cleavage products (cCasp) of caspases 9, 8 and 3 at different time point. Cell lysates were harvested 24 h or 48 h post treatment. Lysates of DMSO-treated cells served as a negative control.
(TIF)

S1 Table Sequences of gRNAs targeting Bad, Noxa and Puma. Complementary gRNA sequences and the corresponding PAMs used for targeting Bad, Noxa and Puma specific exons in non-human primate (NHP) cells using CRISPR/Cas9. The first exon was targeted, except where it was too short for prediction, in which case the second exon was then used. F: forward, R: reverse.
(DOCX)

S2 Table Primers for RT-qPCR amplification of selected apoptosis-related genes. Primers sequences used for the detection of target genes in non-human primate cells (NHP). Amplicon lengths in base pairs (bp) and their specific annealing temperatures (Ta) for quantitative real-time PCR are indicated. F: forward, R: reverse.
(DOCX)
