# Correction: BH3-only sensors Bad, Noxa and Puma are Key Regulators of Tacaribe virus-induced Apoptosis

**DOI:** 10.1371/journal.ppat.1014505

**Published:** 2026-08-13

**Authors:** Julia Holzerland, Lucie Fénéant, Logan Banadyga, Julia E. Hölper, Michael R. Knittler, Allison Groseth

After this article [1] was published, concerns were raised regarding Figs 4,5 and 8. Specifically, that the following panels appear similar to each other:

- The Mock and TCRV BIM α-tubulin panels in Fig 4C.
- The TCRV 4 dpi and Ctrl ind Vinculin bands in Fig 5B.
- The TCRV 4 dpi and TCRV 3dpi Bad panels in Fig 8A.

The corresponding author stated that the TCRV BIM α-tubulin panel in Fig 4C, the Ctrl Ind Vinculin panel in Fig 5B, and the TCRV 4 dpi Bad panel in Fig 8A are incorrect, and provided correct versions of Figs 4,5 and 8 with correct panels from the original experiments.

The original images underlying all panels in Figs 4C, 5B and 8A are included as S1-S3 Files, respectively.

The raw data underlying Figs 2-9 are not provided in the list of Supporting Information. The authors have provided the data as S4 File and at the following repository https://zenodo.org/records/21473001 [2]. With this correction, all relevant data are now provided.

**Fig 4:**
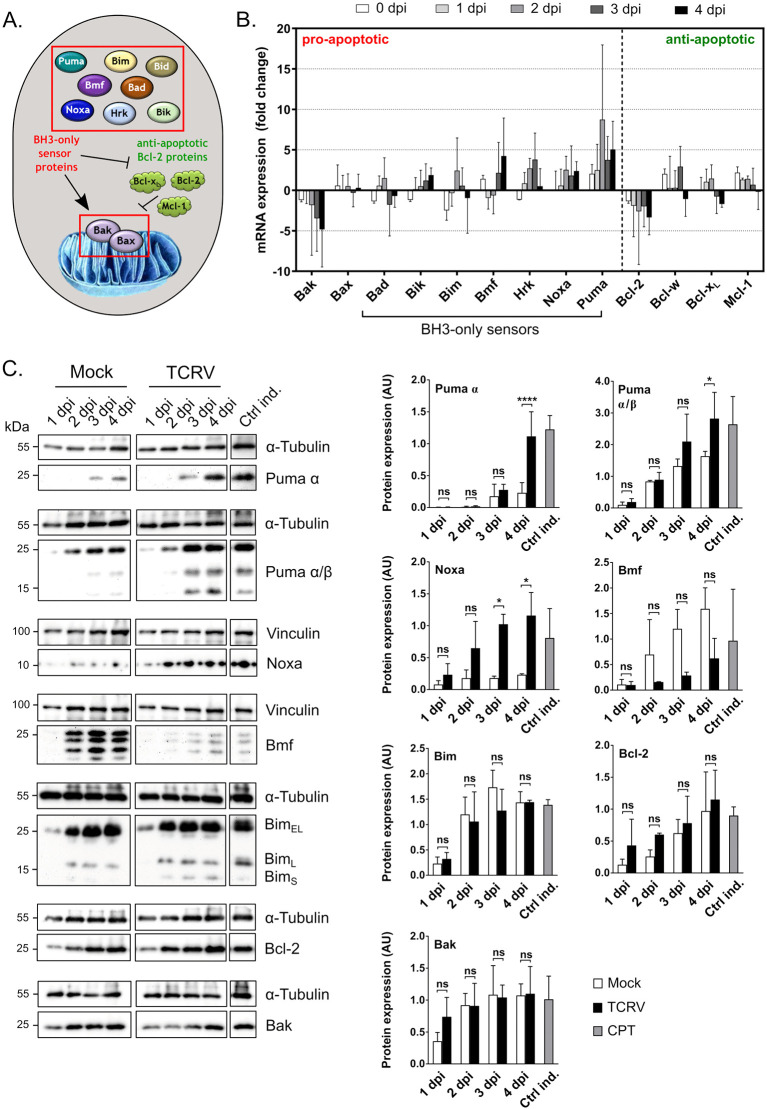
TCRV affects pro- and anti-apoptotic regulators on the mRNA transcript and protein levels. **(A) Schematic model of the host cell factors involved in regulation of mitochondrial permeability.** Pro-apoptotic BH3-only sensors can either directly activate Bak and Bax (both framed in red boxes) or antagonize their inhibitors, the anti-apoptotic Bcl-2 proteins (shown in green). **(B) Transcript levels of pro- and anti-apoptotic regulators of apoptosis.** TCRV infection was performed in Vero76 cells at an MOI of 2 and samples were harvested each day for RNA extraction. mRNA expression levels were determined using RT-qPCR with gene specific primer sets (S1 Table) from 0–4 dpi, as indicated. GAPDH levels were used for standardization and fold change in mRNA levels of TCRV-infected cells (compared to mock-infected cells) was calculated using the 2^-ΔΔCt^ method. Mean values and standard deviations of at least three independent experiments are shown. **(C) Protein levels of selected pro- and anti-apoptotic regulators of apoptosis.** Vero76 cells were infected as above and lysates were subjected to Western blotting with antibodies specific for Puma α/β, Puma α, Noxa, Bmf, Bim, Bcl-2 or Bak, as indicated. Mock cells served as a negative control, while CPT (10 μM) treated cells served as a positive control (Ctrl ind.). Staining for Vinculin or α-Tubulin, were used for loading controls. Western blots were evaluated by measuring pixel intensities for protein bands with normalization to the associated loading control. Quantifications are shown as mean values and standard deviations of at least two independent experiments. Statistical significance was determined using two-way ANOVA (*p ≤ 0.05, ****p ≤ 0.0001, ns not significant).

**Fig 5:**
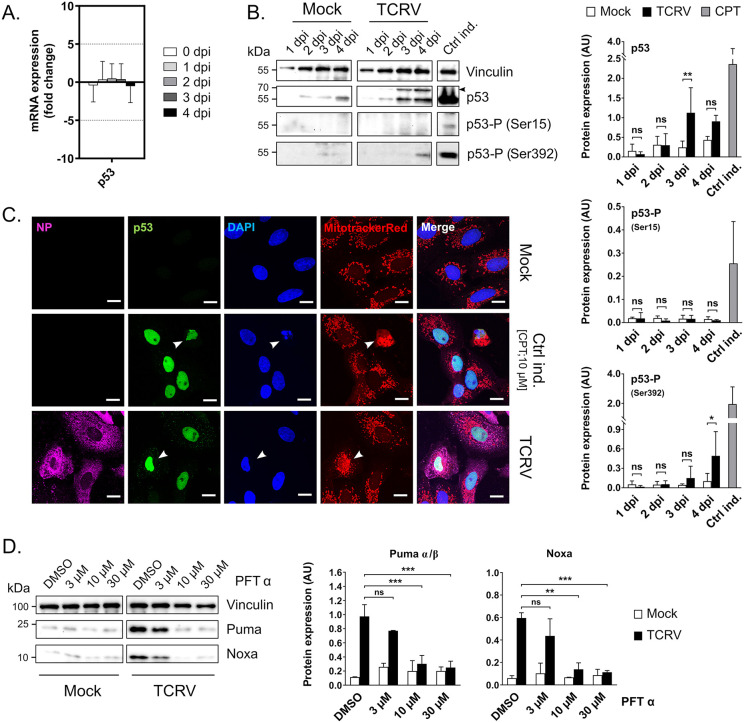
Protein expression, phosphorylation and nuclear translocation of p53 in TCRV-infected cells. **(A) Transcript levels of p53**. TCRV infection was performed in Vero76 cells at an MOI of 2 and samples were harvested each day for RNA extraction. Expression of p53 was quantified using RT-qPCR with a gene specific primer set (S1 Table) at the indicated time points between 0–4 days post infection (dpi). GAPDH levels were used for standardization and fold change in mRNA levels of TCRV-infected cells (compared to mock-infected cells) was calculated using the 2^-ΔΔCt^ method. Values represent the means and standard deviations from three independent replicates. **(B) Protein expression levels and phosphorylation of p53**. Cell lysates from TCRV-infected cells between 1 to 4 dpi were analysed by Western blot for total p53 expression, as well for phosphorylation at Ser15 or Ser392 with the respective antibodies. Vinculin served as a loading control. Mock cells served as a negative control, while CPT treatment (10 μM) was used as a positive control (Ctrl ind.). An arrowhead indicates an unidentified cross-reactive band that is also detected with the anti-p53 antibody. Western blots were evaluated by measuring pixel intensities with normalization to the associated loading control. Quantifications are based on at least two independent experiments. Statistical significance was determined using two-way ANOVA (*p ≤ 0.05, **p ≤ 0.01, ns not significant). (C) p53 nuclear translocation. Mitochondria were stained 3 dpi with TCRV using MitotrackerRed (red), followed by antibody staining using anti-p53 (green) and anti-TCRV NP (magenta), and labeling of nuclei with DAPI (blue). Mock cells served as a negative control, while CPT treatment (10 μM) was used as a positive control (Ctrl ind.). Arrowheads highlight morphological changes consistent with cells in the late stages of apoptosis. Scale bars show a distance of 10 μm. **(D)** Impact of p53 inhibition on Puma and Noxa expression. Vero76 cells were treated daily with PFT-α (3, 10 and 30 μM) or DMSO only and either Mock- or TCRV-infected (MOI = 1). Cell lysates were harvested 4 dpi and analysed for Puma and Noxa expression via Western blot, while Vinculin served as a loading control. Puma and Noxa expression were quantified based on two independent experiments and normalized to the associated loading control. Statistical significance was determined using two-way ANOVA (**p ≤ 0.01, ***p ≤ 0.001, ns not significant).

**Fig 8:**
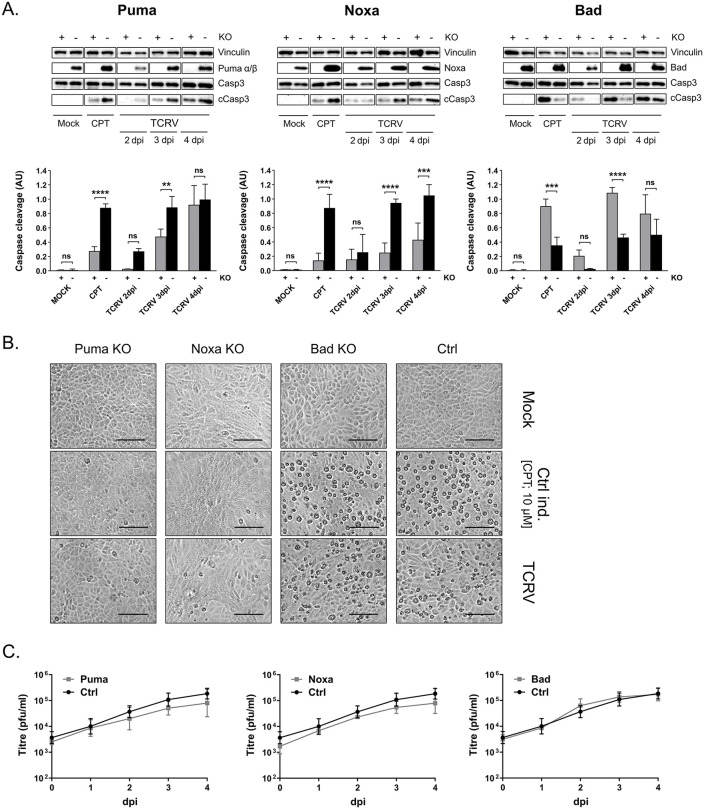
Influence of Puma, Noxa and Bad knockout (KO) on TCRV-induced apoptosis. **(A) BH3-only protein expression and caspase cleavage in KO cells infected with TCRV.** KO cells (+) and control parental cells (-) were infected with TCRV (MOI 1) or mock-infected and lysed 2 to 4 dpi before being analysed by Western blot for Bad, Noxa, Puma, full-length caspase 3 (Casp3) or its cleavage product (cCasp), as indicated, as well as the loading control Vinculin. CPT treatment (10 μM) was used as a positive control. Pixel intensities of cleaved Casp3 protein bands were measured and normalized to full-length Casp3 bands. Quantifications show mean values and standard deviations from at least two independent experiments. Statistical significance was determined using two-way ANOVA (**p ≤ 0.01, ***p ≤ 0.001, ****p ≤ 0.0001, ns not significant). **(B)** CPE formation in KO cells during TCRV infection. Bright field images of KO and parental control cells were taken 4 dpi and are shown with 100 μm scale bars. **(C)** Virus growth in Bad, Noxa and Puma KO cells. Cells were infected with TCRV at an MOI of 1. Supernatants were harvested 0 to 4 dpi and virus titres were determined by plaque assay.

## Supporting information

S1 File Original images underlying all panels in Fig 4C. (ZIP)

S2 File Original images underlying all panels in Fig 5B. (ZIP)

S3 File Original images underlying all panels in Fig 8A. (ZIP)

S4 File Original quantitative data underlying all figures. (ZIP)
