# Supplementary figures and images for: BH3-only sensors Bad, Noxa and Puma are Key Regulators of Tacaribe virus-induced Apoptosis
Source: PLoS Pathog. 2020 Oct 12;16(10):e1008948. doi: 10.1371/journal.ppat.1008948 (PMC7598930; doi:10.1371/journal.ppat.1008948)

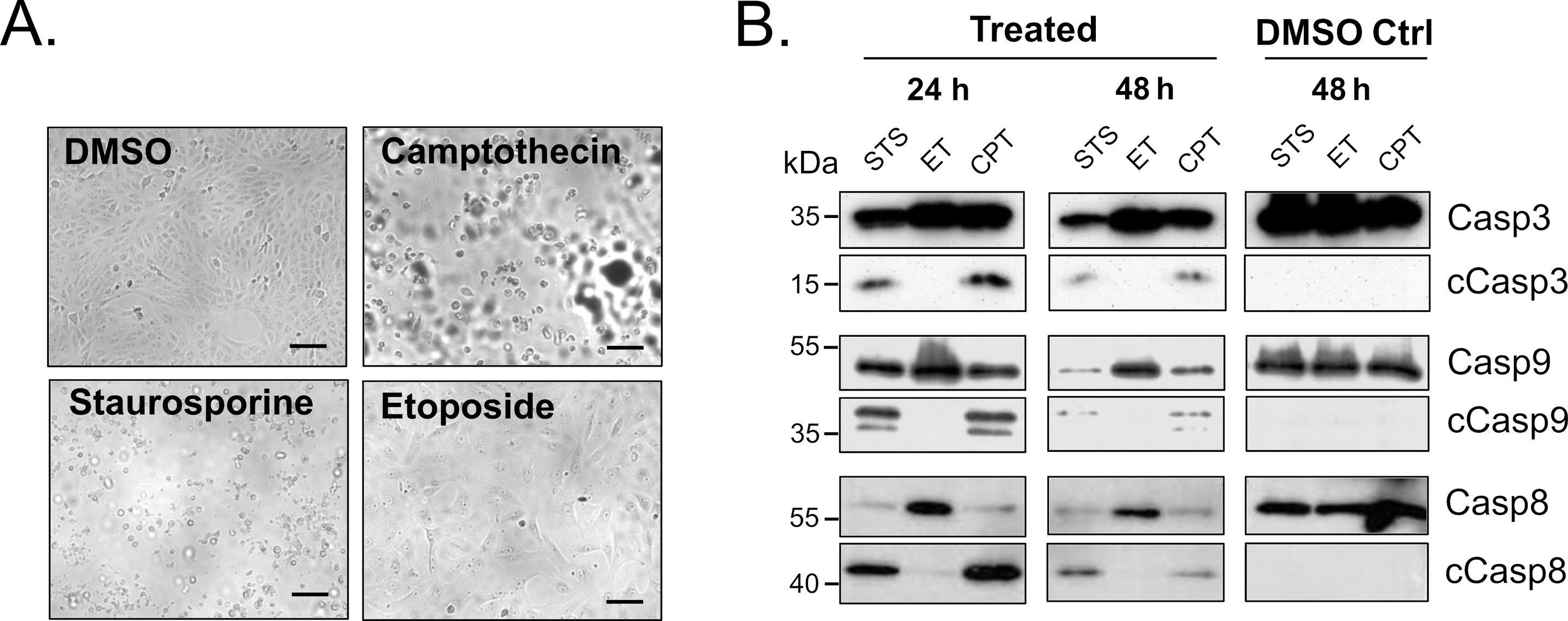

Supplement: S1 Fig — In order to determine suitable doses and incubation times needed to detect caspase activation, Vero76 cells were treated with Camptothecin (CPT; 10 μM), Staurosporine (STS; 1 μM) or Etoposide (ET; 25 μM) for 24 and 48 hours. (A) Microscopic images of induced cell death. Morphological changes in treated cells compared to a DMSO control treatment (top picture) 48 h post treatment. Scales bars show distances of 100 μm. (B) Detection of caspase cleavage in treated cells. Western blot analyses were performed to investigate caspase activation using antibodies against full-length (Casp) and cleavage products (cCasp) of caspases 9, 8 and 3 at different time point. Cell lysates were harvested 24 h or 48 h post treatment. Lysates of DMSO-treated cells served as a negative control. (TIF) [file ppat.1008948.s001.tif]
