# Supplementary material for: BH3-only sensors Bad, Noxa and Puma are Key Regulators of Tacaribe virus-induced Apoptosis
Source: PLoS Pathog. 2020 Oct 12;16(10):e1008948. doi: 10.1371/journal.ppat.1008948 (PMC7598930; doi:10.1371/journal.ppat.1008948)
Supplement: S1 Table — Complementary gRNA sequences and the corresponding PAMs used for targeting Bad, Noxa and Puma specific exons in non-human primate (NHP) cells using CRISPR/Cas9. The first exon was targeted, except where it was too short for prediction, in which case the second exon was then used. F: forward, R: reverse. (DOCX) [file ppat.1008948.s002.docx]

| Target gene | gRNA | Exon | Full length target sequence 5‘ → 3‘ | PAM |
| --- | --- | --- | --- | --- |
| Puma | #1 | 1 | F: ATG GCC CGC GCA CGC CAG GA  R: TCC TGG CGT GCG CGG GCC AT | GGG |
| Puma | #2 | 1 | F: AGC TCC CCG GAG CCC GTA GA  R: TCT ACG GGC TCC GGG GAG CT | GGG |
| Puma | #3 | 1 | F: GTA GAG GGC CTG GCC CGC GA  R: TCG CGG GCC AGG CCC TCT AC | CGG |
| Puma | #4 | 1 | F: AGC TGC CCT CCT GGC GTG CG  R: CGC ACG CCA GGA GGG CAG CT | CGG |
| Noxa | #1 | 1 | F: ACG CGC AAC CGA GCC CAA CG  R: CGT TGG GCT CGG TTG CGC GT | CGG |
| Noxa | #2 | 1 | F: ACC GAG CCC AAC GCG GGC TC  R: GAG CCC GCG TTG GGC TCG GT | AGG |
| Noxa | #3 | 1 | F: AGC CCA ACG CGG GCT CAG GC  R: GCC TGA GCC CGC GTT GGG CT | CGG |
| Noxa | #4 | 1 | F: GGG AAG AAG GCG CGC AAG AA  R: TTC TTG CGC GCC TTC TTC CC | AGG |
| Bad | #1 | 2 | F: CTC CGG CAA GCA TCA TCA CC  R: GGT GAT GAT GCT TGC CGG AG | AGG |
| Bad | #2 | 2 | F: AGA GTT TGA GCC TAG TGA GC  R: GCT CAC TAG GCT CAA ACT CT | AGG |
| Bad | #3 | 2 | F: GTG GGA CGC CAG TCA CCA GC  R: GCT GGT GAC TGG CGT CCC AC | AGG |
| Bad | #4 | 2 | F: CCT GTG GGA CGC CAG TCA CC  R: GGT GAC TGG CGT CCC ACA GG | AGG |
