# Supplementary material for: BH3-only sensors Bad, Noxa and Puma are Key Regulators of Tacaribe virus-induced Apoptosis
Source: PLoS Pathog. 2020 Oct 12;16(10):e1008948. doi: 10.1371/journal.ppat.1008948 (PMC7598930; doi:10.1371/journal.ppat.1008948)
Supplement: S2 Table — Primers sequences used for the detection of target genes in non-human primate cells (NHP). Amplicon lengths in base pairs (bp) and their specific annealing temperatures (Ta) for quantitative real-time PCR are indicated. F: forward, R: reverse. (DOCX) [file ppat.1008948.s003.docx]

Suppl Table 2. Primers for BH3-only Protein RT-qPCR

| Target | 5‘ → 3‘ sequence | Ta (°C) | Amplicon length (bp) | Reference |
| --- | --- | --- | --- | --- |
| Bax | F: CCC TTT TGC TTC AGG GTT TC  R: TCT TCT TCC AGA TGG TGA GCG | 54 | 501 | [97] |
| Bad | F: CAT CAT GGA GGC GCT GGG GC  R: TG CCG CAT CTG CGT CGC TGT GCC | 65 | 251 | [98] |
| Bak | F: TTT TCC GCA GCT ACG TTT TT  R: TGG TGG CAA TCT TGG TGA AGT | 51 | 249 | [97] |
| Bik | F: CTT GAT GGA GAG CCT CCT GTA TG  R: AGG GTC CAG GTC CTC TTC AGG | 57 | 91 | [99] |
| Bim_EL_ | F: TTC CAT GAG GCA GGC TGA AC  R: CCT CC TTG CAT AGT AAG CGT T | 51 | 103 | [100] |
| Bmf | F: ATG GAG CCA TCT CAG TGT GTG  R: CCC CGT TCC TGT TCT CTT CT | 54 | 541 | [101] |
| Hrk | F: GGC AGG CGG AAC TTG TAG GAA C  R: TCC AGG CGC TGT CTT TAC TCT CC | 57 | 197 | [102] |
| Bcl-2 | F: CTG TGG TCC ACC TGA CCC TCC GC  R: CGT ACA GTT CCA CAA AGG CGT CCC AGC | 65 | 336 | [98] |
| Bcl-w | F: AGT TCG AGA CCC GCT TCC  R: CCC GTC CCC GTA TAG AGC | 54 | 308 | [97] |
| Bcl-x_L_ | F: CTG AAT CGG AGA TGG AGA CC  R: TGG GAT GTC AGG TCA CTG AA | 51 | 211 | [97] |
| Mcl-1 | F: AGA AAG CTG CAT CGA ACC AT  R: CC AGC TCC TAC TCC AGC AAC | 54 | 183 | [97] |
| Noxa | F: AGC TGG AAG TCG AGT GTG CT  R: ACG TGC ACC TCC TGA GAA AA | 54 | 167 | [97] |
| Puma | F: GGA GCA GCA CCT GGA GTC  R: TA CTG TGC GTT GAG GTC GTC | 54 | 156 | [97] |
| p53 | F: AGG CCT TGG AAC TCA AGG AT  R: TGA GTC AGG CCC TTC TGT CT | 54 | 140 | [103] |
| GAPDH | F: CCA GGT GGT CTC CTC TGA CTT CAA  R: ATA CCA GGA AAT GAG CTT GAC A | 54 | 106 | [103] |
