# Supplementary material for: Correction: BH3-only sensors Bad, Noxa and Puma are Key Regulators of Tacaribe virus-induced Apoptosis
Source: PLoS Pathog. 2026 Aug 13;22(8):e1014505. doi: 10.1371/journal.ppat.1014505 (PMC13472383; doi:10.1371/journal.ppat.1014505)
Supplement: Supplementary file 4 [file ppat.1014505.s004.zip › S4 File/Figs 2 and 7 FACS/Explanation of the Compensation Files.docx]

Explanation of the Compensation Files

Fluorescence spillover between channels was corrected by post-acquisition compensation using the MACSQuant-integrated MACSQuantify™ compensation 7 × 7 channel matrix tool and applied to the analyzed samples to generate compensated datasets. The compensation/spillover value for the FITC/B3 matrix cell was set to **0.065** to diminish spillover between the two channels.
